# Spatial mapping of the HCC landscape identifies unique intratumoral perivascular-immune neighborhoods

**DOI:** 10.1097/HC9.0000000000000540

**Published:** 2024-10-17

**Authors:** Felix Marsh-Wakefield, Cositha Santhakumar, Angela L. Ferguson, Thomas M. Ashhurst, Joo-Shik Shin, Fiona H.X. Guan, Nicholas J. Shields, Barry J. Platt, Givanna H. Putri, Ruta Gupta, Michael Crawford, Carlo Pulitano, Charbel Sandroussi, Jerome M. Laurence, Ken Liu, Geoffrey W. McCaughan, Umaimainthan Palendira

**Affiliations:** 1 Liver Injury & Cancer Program, Centenary Institute, Camperdown, New South Wales, Australia; 2 Human Immunology Laboratory, School of Medical Sciences, Faculty of Medicine and Health, The University of Sydney, Camperdown, New South Wales, Australia; 3 A.W. Morrow Gastroenterology and Liver Centre, Royal Prince Alfred Hospital, Camperdown, New South Wales, Australia; 4 School of Medical Sciences, Faculty of Medicine and Health, The University of Sydney, Camperdown, New South Wales, Australia; 5 Sydney Cytometry Core Research Facility, The University of Sydney, Camperdown, New South Wales, Australia; 6 Central Clinical School, Sydney Medical School, The University of Sydney, Camperdown, New South Wales, Australia; 7 Department of Tissue Pathology and Diagnostic Oncology, Royal Prince Alfred Hospital, NSW Health Pathology, Camperdown, New South Wales, Australia; 8 The Walter and Eliza Hall Institute of Medical Research and The Department of Medical Biology, The University of Melbourne, Parkville, Victoria, Australia; 9 Australian National Liver Transplant Unit, Royal Prince Alfred Hospital, Sydney, New South Wales, Australia; 10 Royal Prince Alfred Institute of Academic Surgery, University of Sydney, Camperdown, New South Wales, Australia

**Keywords:** tumour immunology, spatial proteomics, imaging mass cytometry, human liver cancer, perivascular macrophages

## Abstract

**Background:**

HCC develops in the context of chronic inflammation; however, the opposing roles the immune system plays in both the development and control of tumors are not fully understood. Mapping immune cell interactions across the distinct tissue regions could provide greater insight into the role individual immune populations have within tumors.

**Methods:**

A 39-parameter imaging mass cytometry panel was optimized with markers targeting immune cells, stromal cells, endothelial cells, hepatocytes, and tumor cells. We mapped the immune landscape of tumor, invasive margin, and adjacent nontumor regions across 16 resected tumors comprising 144 regions of interest. X-shift clustering and manual gating were used to characterize cell subsets, and Spectre quantified the spatial environment to identify cellular neighborhoods. Ligand-receptor communication was quantified on 2 single-cell RNA-sequencing data sets and 1 spatial transcriptomic data set.

**Results:**

We show immune cell densities remain largely consistent across these 3 regions, except for subsets of monocyte-derived macrophages, which are enriched within the tumors. Mapping cellular interactions across these regions in an unbiased manner identifies immune neighborhoods comprised of tissue-resident T cells, dendritic cells, and various macrophage populations around perivascular spaces. Importantly, we identify multiple immune cells within these neighborhoods interacting with VEGFA^+^ perivascular macrophages. *VEGFA* was further identified as a ligand for communication between perivascular macrophages and CD34^+^ endothelial cells.

**Conclusions:**

Immune cell neighborhood interactions, but not cell densities, differ between intratumoral and adjacent nontumor regions in HCC. Unique intratumoral immune neighborhoods around the perivascular space point to an altered landscape within tumors. Enrichment of VEGFA^+^ perivascular macrophages within these tumors could play a key role in angiogenesis and vascular permeability.

## Graphical abstract

**Figure:**
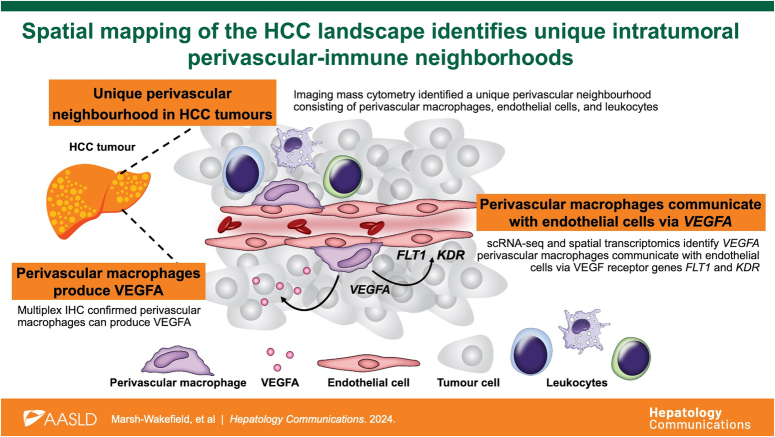

## INTRODUCTION

Liver cancer is the sixth most incident cancer and the third leading cause of cancer-related death in the world,^1^ with HCC accounting for ~90% of cases.^2^ The 5-year overall survival of HCC after diagnosis is ~20%, and this statistic has not changed significantly over the past decade despite advancements in treatment options.^3^ This poor prognosis is attributable to its phenotypic and genetic heterogeneity, its occurrence in patients with reduced liver reserve (cirrhosis), high rates of tumor recurrence following curative treatments, and its predisposition to metastasize in advanced stages.^4^ Although immunotherapy has recently revolutionized the treatment of advanced-stage disease in other malignancies such as melanoma and lung cancer, objective response rates to systemic combination therapies in advanced HCC have been suboptimal (<30%).^5^,^6^,^7^

Being the prototypical cancer of inflammatory origin, immune responses in HCC are implicated in both protumoral and antitumoral activities.^8^,^9^ Understanding the complex interplay between immune cells and the tumor environment is therefore critical. Recent studies using single-cell approaches have identified the diverse phenotype of immune cells within HCC tumors and provided some insight into their possible functional roles.^10^ However, several of these single-cell methodologies lack spatial context, therefore limiting our ability to determine the potential impact that specific cellular interactions have on clinical outcomes. Spatially resolving these cellular interactions could provide greater insight into how various immune populations contribute to tumor development, progression, and control.

Angiogenesis is also a key feature of HCC, wherein hypervascularization and leaky vessels pose challenges to the cellular immune control of tumors and influence responses to various therapies.^11^,^12^,^13^ Among many angiogenic factors, VEGFA has been identified as the key driver of neovascularization in HCC, with tumor cells largely implicated in its production.^14^,^15^ The importance of angiogenesis (and its interplay with the tumor immune microenvironment [TME]) is evident by recent data showing that a combination of bevacizumab (anti-VEGFA) and atezolizumab (anti-programmed cell death ligand 1 [PD-L1]) antibodies improved survival in patients with advanced unresectable HCC.^5^ Interestingly, this strategy has been found to have a synergistic effect.^16^,^17^

HCC tumor cells are a well-known source of VEGFA,^14^,^18^ yet several immune cells have been identified as alternate sources of VEGFA. *VEGFA* ^+^ mast cells are thought to be protumor across several cancer types, although they are largely absent in HCC.^19^ In contrast, high levels of *FOLR2* ^+^ macrophages, *PLVAP* ^+^ endothelial cells, and Notch/VEGF signaling are associated with an immunosuppressive niche within HCC tumors.^20^

High-dimensional imaging mass cytometry (IMC) has previously been utilized to investigate the tumor microenvironment in HCC across several contexts, including HBV infection,^21^ response to immunotherapies,^22^,^23^ and macrophage characterization.^24^ The latter study is important as several studies have implicated macrophages in HCC, including neighborhood interactions associated with immune cell activation^25^,^26^ and response to immunotherapies.^22^,^23^ However, more work is needed to better understand the role of immune cells—particularly macrophages—in angiogenesis and vascular permeability in HCC.

In this study, we interrogated the tumor and adjacent non-TME in treatment-naïve patients with resectable HCC using a high-dimensional tissue imaging platform known as IMC. To date, only 2 other studies have used IMC to investigate the TME in treatment-naïve patients with HCC.^24^,^27^ Our study specifically examined immune (CD45^+^) cell interactions and did not consider cell-cell interactions with the tumor cells themselves. We did this deliberately as it was a novel approach using IMC to provide a more in-depth characterization of these cells and their role(s) in the HCC tumor microenvironment. Important cellular interactions within the CD45^+^ populations could be overshadowed or lost if we included cell-to-cell interactions with cancer cells themselves. This targeted approach identified over 10,000 parameters for each patient and led to the novel identification of perivascular-immune neighborhoods within HCC tumor tissue that we suggest may have gone unnoticed otherwise. Specifically, we showed enrichment of VEGFA^+^ perivascular macrophages (PVMs) within the tumor regions that interact with these immune aggregates in the perivascular space that was absent in adjacent nontumor regions, suggesting these immune neighborhoods could be important in the development and/or progression of HCC.

## METHODS

### Patients

Ethics was obtained by the Sydney Local Health District Ethics Review Committee (HREC 2020/ETH02093). Sixteen treatment-naïve patients were undergoing curative resection for HCC. Formalin-fixed paraffin-embedded tissues were retrieved with clinicopathological data summarized in Supplemental Table S1, http://links.lww.com/HC9/B51 and Supplemental Methods, http://links.lww.com/HC9/B51.

### Tissue microarray preparation

**FIGURE 1:**
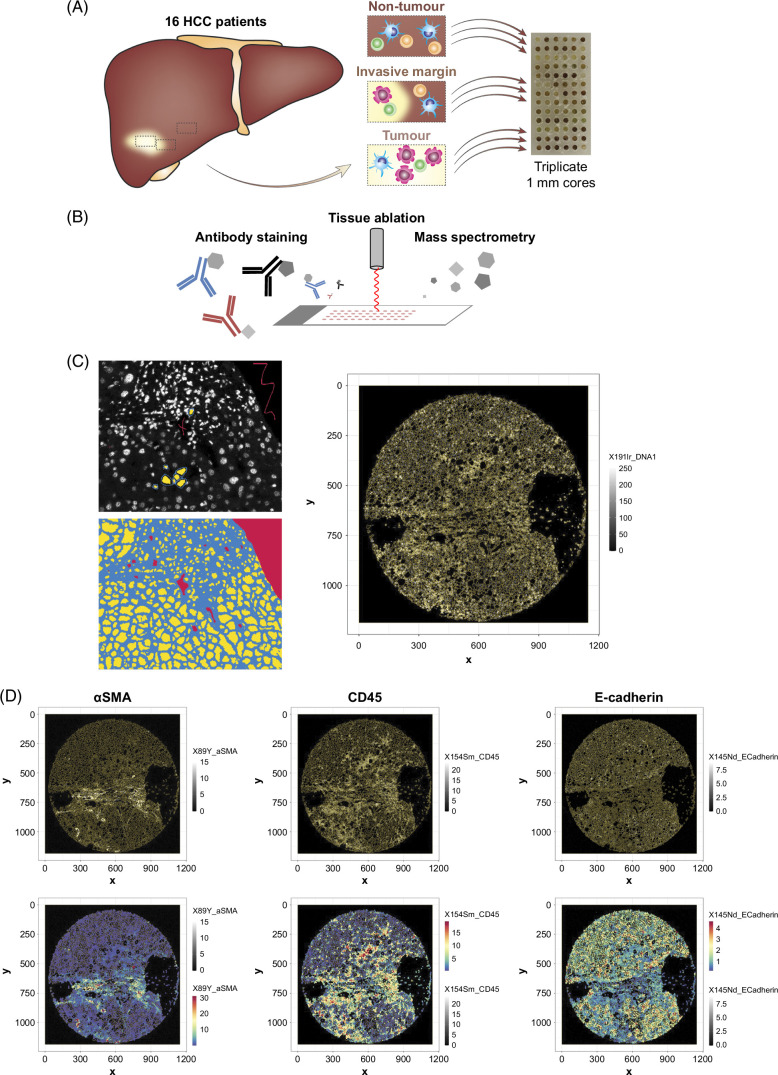
Experimental overview. (A) Sixteen patients with HCC underwent liver resections. One millimeter diameter regions of nontumor, invasive margin, and tumor were selected from each patient in triplicate to create a tissue microarray. (B) Sections were stained with antibodies conjugated to heavy metals which were then ablated by a Helios imaging mass cytometer to quantify regions. (C) Ilastik software was used to define cell borders to create a mask of single cells. (D) R package Spectre quantified marker expression for each segmented cell across regions.

Hematoxylin and eosin sections were reviewed by a liver pathologist. One region of interest was selected from the tumor, invasive margin, and nontumor regions. Three 1 mm diameter cores were taken from each region of interest for the creation of the 2 tissue microarrays. Figure 1 provides an overview of the experimental design.

### Antibody panel

Antibodies were previously validated in our lab on human tissue.^28^ Antibodies were then revalidated on formalin-fixed paraffin-embedded liver and HCC tissue (Supplemental Table S2, http://links.lww.com/HC9/B51, Supplemental Figure S1, http://links.lww.com/HC9/B51).

### IMC

The Hyperion imaging mass cytometer (Standard BioTools) acquired data that were analyzed using MCD Viewer (version 1.0.560.6, Standard BioTools) and histoCAT++ (version 2.2)^29^ (Figures 1C, D). Segmented IMC data are available on Zenodo (https://zenodo.org/records/10622397).

### Multiplex immunohistochemistry

Four micrometers formalin-fixed paraffin embedded sections were stained using an OPAL multiplex immunohistochemistry (mIHC) staining kit (Akoya Biosciences) as described.^30^,^31^ Images were captured using Mantra Snap (version 1.0.3, Akoya Biosciences) and inForm (version 2.4.2, Akoya Biosciences) and processed in Fiji (version 1.53c).^32^ Cell segmentation, phenotyping, and spatial analysis were done in HALO (version 3.6.4134, Indica Labs).

### Single-cell transcriptomic analyses

Cell segmentation using multicut was done in Ilastik (version 1.4.0b27)^33^ before marker quantification in R (version 4.2.0)^34^ using “Spectre” (version 1.0 and development version)^35^ (Figure 1D). Region of interest area was calculated using Fiji (version 1.53c).^32^

Single cells were manually gated using FlowJo (version 10.8, BD Bioscience) (Supplemental Figure S3, http://links.lww.com/HC9/B51). X-shift clustering (version 26-Apr-2018)^36^ was used in conjunction with “clustsig” (version 1.1).^37^ Spatial data were quantified using Spectre.^35^ “mixOmics” was used for the sparse partial least squares-discriminant analysis (sPLS-DA) (version 6.24.0).^38^,^39^

Preprocessed and preannotated single-cell RNA-sequencing (scRNA-seq) data from the GSE149614 data set (https://www.ncbi.nlm.nih.gov/geo/query/acc.cgi?acc=GSE149614) were used.^40^ A second cohort of HCC patients was analyzed (https://data.mendeley.com/datasets/skrx2fz79n/1).^41^ Cluster annotations from the first cohort were transferred to the second cohort using “scANVI” (“scvi-tools,” version 1.0.4)^42^,^43^ (Supplemental Figure S5, http://links.lww.com/HC9/B51). Ligand-receptor communication was calculated using “CellCall” (version 1.0.7).^44^ A segmented and annotated CosMx SMI data set from nanoString was analyzed using the vignette.^45^

### Statistical tests

Statistics were calculated using R and GraphPad Prism (version 9.0.0). Permutational tests are powerful nonparametric tests that do not assume homogeneity of variance or normality of distribution.^46^ R packages “vegan” (version 2.6-4),^47^ “pairwiseAdonis” (version 0.4.1),^48^ and “RVAideMemoire” (version 0.9-83)^49^ were used. Plots were generated in R and GraphPad Prism.

## RESULTS

### The spatial landscape differs between HCC tumor regions

**FIGURE 2:**
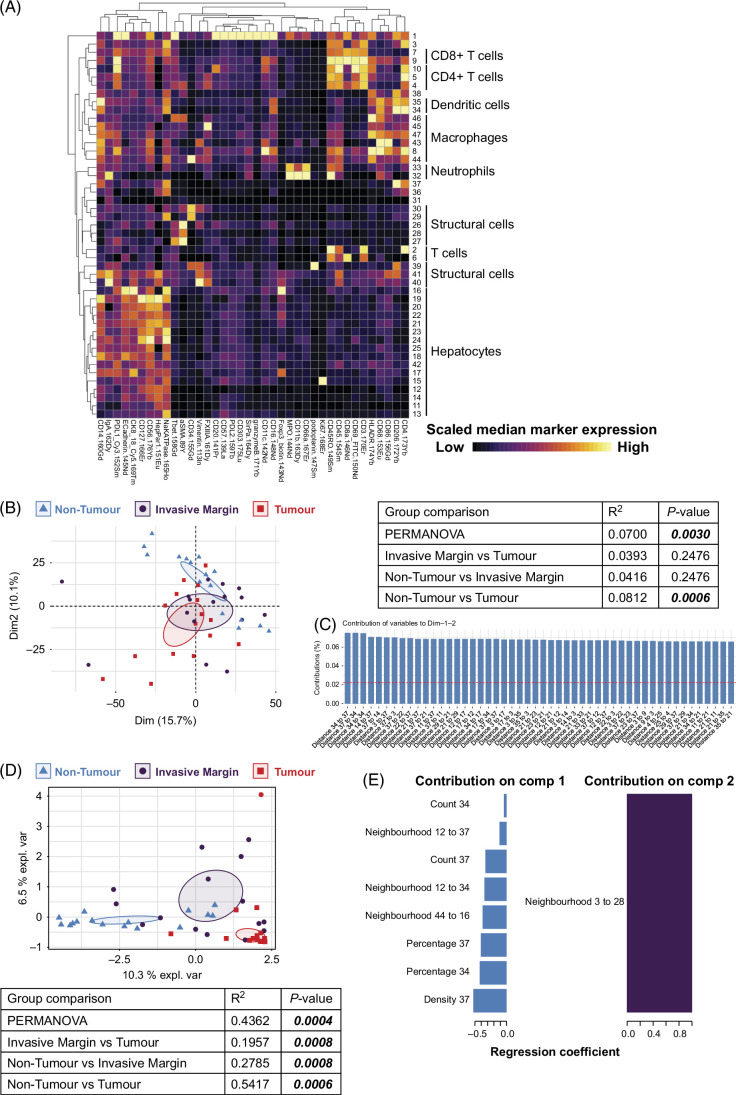
Spatial environment differs between liver regions. (A) X-shift clustering was used to identify 47 clusters represented by a heatmap. Each row is a different cluster, and each column is a different marker. (B) PCA of all spatial data. Symbols and colors represent a different region from individual patients. Ninety-five percent CI shown. A PERMANOVA with Holm correction was performed on all parameters. (C) The parameters that contributed most to the variance across the first 2 components are shown. Horizontal red dashed line represents the value if all parameters contributed equally. (D) An sPLS-DA generated a similar plot, with the selected parameters for the first 2 components shown. A PERMANOVA with Holm correction was performed on select parameters from the first 2 components. (E) The parameters that contribute most to the differences between regions across the first 2 components are shown. n = 16 patients with HCC, each with nontumor, invasive margin, and tumor regions. Abbreviations: PCA, principal component analysis; PERMANOVA, permutational multivariate analysis of variance; sPLS-DA, sparse partial least squares-discriminant analysis.

We mapped the full cellular landscape (immune cells, stromal cells, endothelial cells, hepatocytes, and tumor cells) of 16 treatment-naïve patients with HCC to identify differences between nontumor, invasive margin, and tumor regions (Figures 1A, B). Following cell segmentation (Figure 1C) and protein quantification (Figure 1D), X-shift clustering was performed on cells that identified 47 clusters (Figure 2A). Cell segmentation is problematic when defining single cells sectioned from a 3-dimensional organ, resulting in some single cells being clustered together (Supplemental Figure S6, http://links.lww.com/HC9/B51). The tumor spatial environment was quantified for each patient and tissue region. A principal component analysis was run on >10,000 parameters, including cluster levels (density, percentage of total cells, and count), average distances between clusters, and neighborhood interactions across each region. Cellular neighborhoods were inferred based on the average number of 1 cluster within 20 μm of another (or the same) cluster.^50^ The permutational multivariate analysis of variance revealed significant differences between the 3 regions (*p* = 0.0030), which was limited to a difference between nontumor and tumor (*p* = 0.0006) but not between invasive margin and tumor or nontumor (Figure 2B, *p* = 0.2467). We then determined the parameters that contribute most to the identified variance. This revealed the primary differentiator as the distance between individual clusters, suggesting the spatial distribution of cells could be key in HCC tumor tissue (Figure 2C). A principal component analysis reduces dimensions based on total variance and will be affected by human and experimental variance. In contrast, an sPLS-DA reduces dimensions based on differences between groups.^38^ The sPLS-DA showed clear differences between each of the 3 regions (Figure 2D). The differentiating parameters included not only the distance between clusters, but also cell proportions and densities (Figure 2E). Component 1 (represented by the *x*-axis) separated all regions (Figure 2E). Cluster 37, identified as endothelial cells, had differences in density, percentage, and count. Cluster 34, another endothelial cell subset, differed in percentage and count. Cellular neighborhood interactions of cluster 44 (myeloid cells) to 16 (hepatocytes), cluster 12 (hepatocytes) to 34 (endothelial cells), and cluster 12 (hepatocytes) to 37 (endothelial cells) also differed between regions, particularly between nontumor and tumor regions. Component 2 (*y*-axis) separated the invasive margin (Figure 2E), with cellular neighborhood interactions of cluster 3 (T cells) to 28 (endothelial cells). Together, these data show that our unbiased analytical approach to identify differences in HCC tissue regions demonstrates clear distinctions in the overall cellular landscape. We were particularly interested in the immune cell microenvironment within these regions. Thus, we specifically examined the quantities and spatial relationships of immune cells.

### Innate, but not adaptive, immune cell densities differ between HCC and adjacent tissue

**FIGURE 3:**
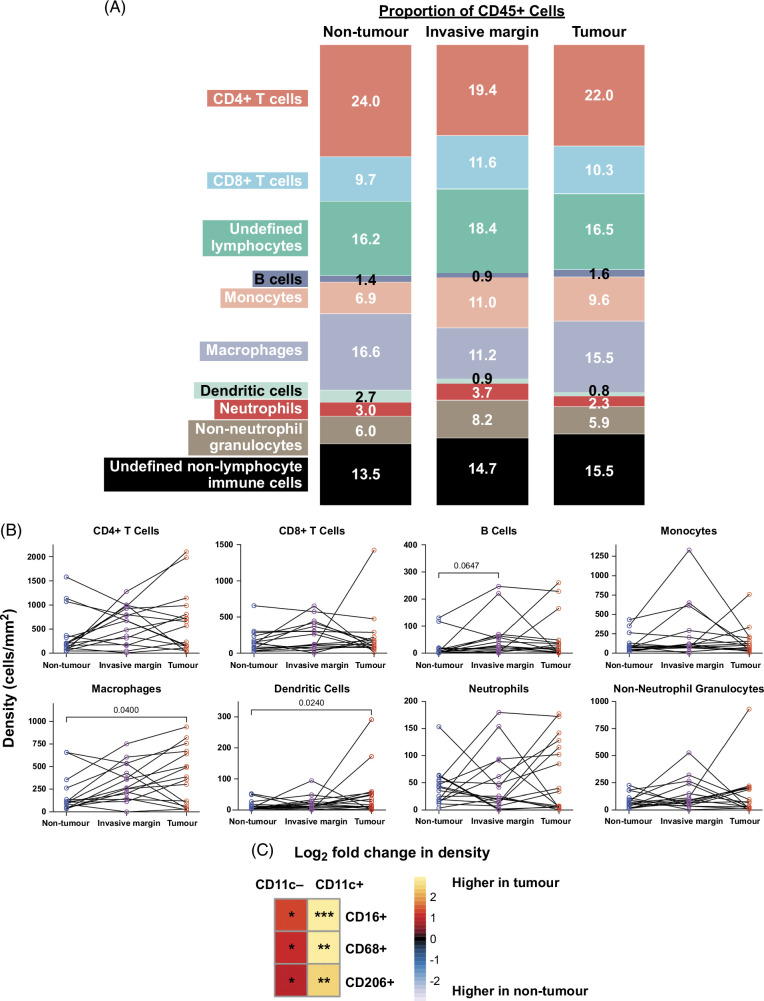
Tumor has higher densities of monocyte-derived CD11c^+^ and CD11c^–^ macrophage subsets compared to nontumor regions. (A) Proportion of conventional immune cells across regions. (B) The density of conventional immune subsets across regions. Friedman test with the Dunn multiple comparison corrections was performed. (C) Log_2_ fold change comparing densities of monocyte-derived CD11c^+^ and CD11c^–^ macrophages between tumor and nontumor regions. Wilcoxon test was used. *p < 0.05, **p < 0.01, ***p < 0.001. n = 16 patients with HCC.

Differences in the immune landscape of the tumor, invasive margin, and adjacent nontumor regions were investigated by manual gating (Figure 3). Surprisingly, the densities of immune subsets were mostly similar across the 3 regions. T cells (particularly CD4^+^ T cells) constituted the largest proportion of immune cells across all regions and their densities remained consistent (Figures 3A, B, Supplemental Figure S7A, http://links.lww.com/HC9/B51). Of the myeloid cells, macrophages constituted the largest proportion across regions (Figure 3A). There was a higher density of macrophages and dendritic cells within tumor compared to nontumor regions (Figure 3B).

A more targeted analysis of innate immune cell subsets revealed subsets of CD14^+^ macrophages (CD16^+^ CD68^+^ CD206^+^ CD11c^+/–^) were significantly higher within tumor compared with nontumor regions (Figure 3C). In contrast, CD11c^+^ and CD11c^–^ macrophages that were CD14^–^ were not different (Supplemental Figure S7B, http://links.lww.com/HC9/B51). There was a higher proportion of these subsets within the tumor compared with nontumor regions, except for monocyte-derived CD206^+^ CD11c^–^ macrophages (Supplemental Figure S7C, http://links.lww.com/HC9/B51). As a proportion of total immune cells, only monocyte-derived CD11c^+^ macrophages were higher in the tumor compared with nontumor regions (Supplemental Figure S7C, http://links.lww.com/HC9/B51).

There were no significant differences in the density of T-cell subsets between regions (Figure 3B). Most T cells expressed CD4^+^ and CD69, consistent with tissue-resident CD4^+^ T cells (Supplemental Figure S7A, http://links.lww.com/HC9/B51). Together, these data demonstrate that innate immune cells, particularly monocyte-derived macrophages, are significantly increased within tumor compared to nontumor regions.

### Unique immune cell neighborhood interactions exist within the HCC tumor

**FIGURE 4:**
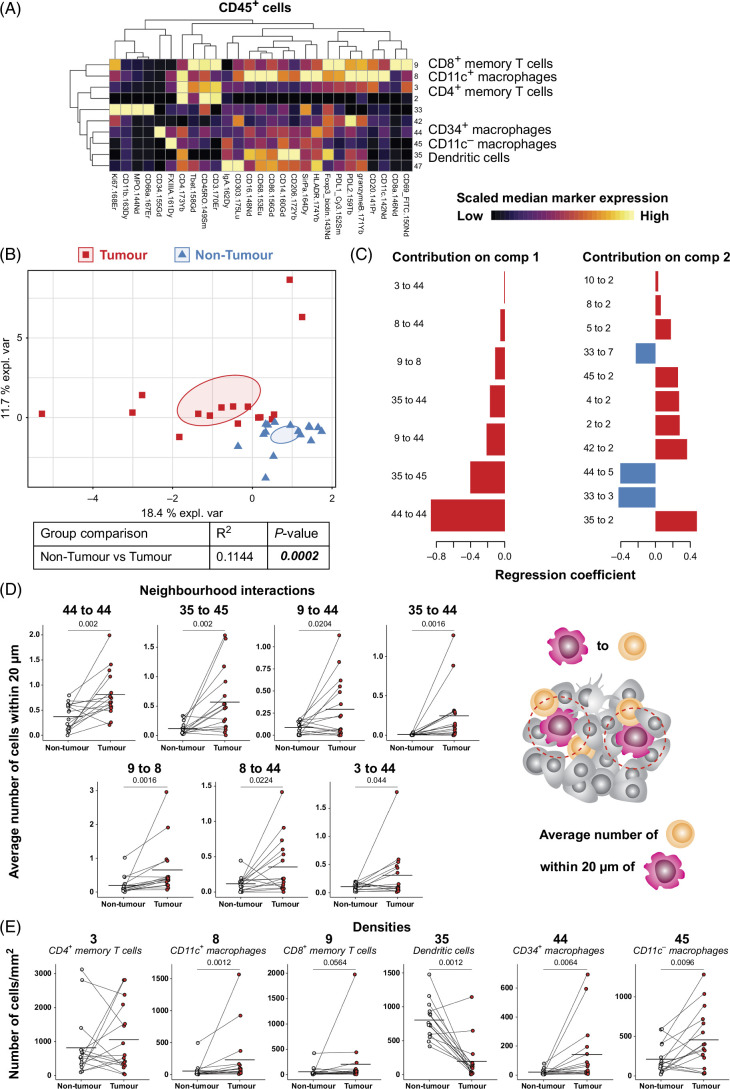
Immune cell neighbors differ between nontumor and tumor regions. (A) Heatmap of CD45^+^ clusters. Each row is a cluster, and each column is a marker. (B) sPLS-DA comparing nontumor to tumor regions. Symbols and colors represent a different region from individual patients. Ninety-five percent CI shown. (C) Selected parameters for the first 2 components are shown. Select comparisons between nontumor and tumor cellular neighborhood interactions (D) and densities (E). n = 16 patients with HCC, each with nontumor, invasive margin, and tumor regions. A permutation Student *t* test was done, *p* values ≤0.1 are shown, and mean is shown.

As the densities and proportions of most conventional immune cell subsets were similar across regions, we next spatially analyzed these regions to investigate immune cell neighborhood interactions. When investigating CD45^+^ clusters alone to focus on immune cells, 6 clusters had <5 cells in more than one-third of the images, so were removed from further analysis (Supplemental Figure S4D, http://links.lww.com/HC9/B51). Furthermore, certain clusters were phenotypically similar based on marker expression, so were combined (Supplemental Figure S6, http://links.lww.com/HC9/B51). As a result, 10 CD45^+^ clusters were included in the neighborhood analysis (Figure 4A).

To identify immune cell interactions that differ between regions, an sPLS-DA was performed, which revealed differences in immune cell neighborhood interactions between nontumor and tumor regions (Figure 4B). Multiple immune cell neighborhood interactions were identified (Figure 4C). The first component (*x*-axis) parameters were further investigated. Cluster 44 was more commonly within 20 μm of other clusters (including 3, 8, 9, 35, and 44) within tumor compared to nontumor regions. Similarly, cluster 35 was commonly within 20 μm of cluster 45, and cluster 9 within 20 μm of cluster 8 within HCC tumor regions (Figure 4D). We then determined whether these interactions could have been impacted by individual cell densities. The density of clusters 8, 9, 44, and 45 was higher within tumor compared to nontumor whereas the opposite was true for cluster 35 (Figure 4E). There was no difference in the density of cluster 3 when comparing tumor and nontumor regions. These data show that multiple immune cell neighborhood interactions are more frequent within HCC tumor regions when compared to nontumor regions. Some of these increased cellular neighborhood interactions may be attributed to increased densities of the cells involved.

### Increased immune cell interactions with VEGFA^+^ PVMs in HCC tumor tissue

**FIGURE 5:**
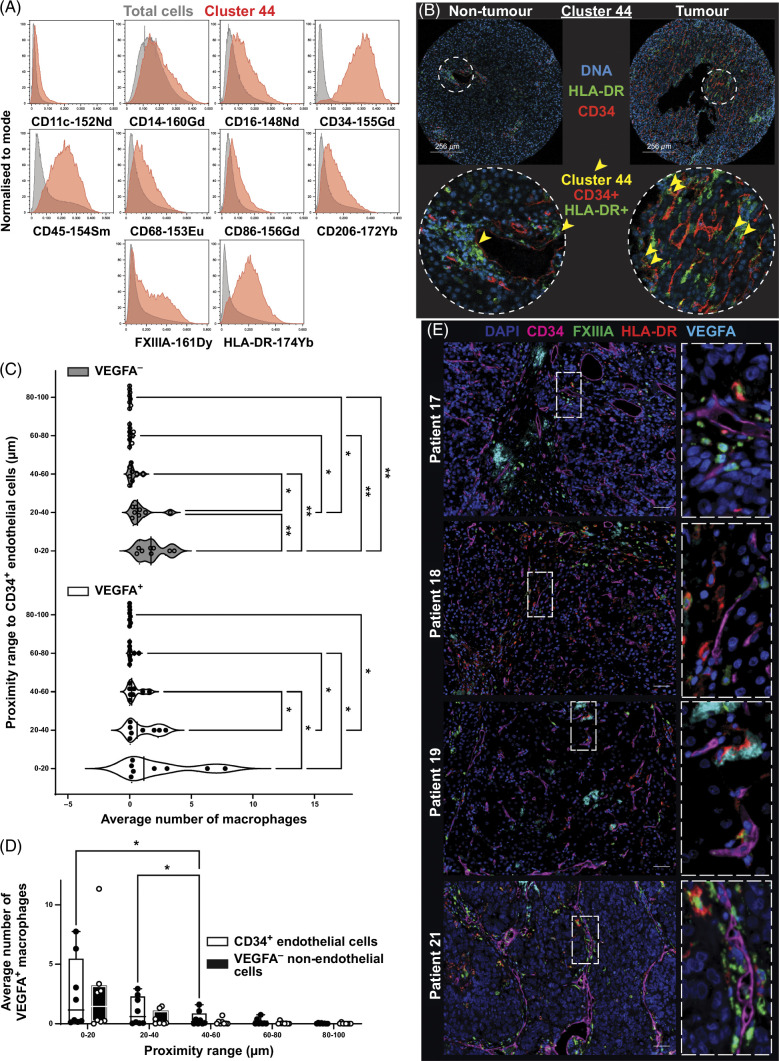
Cluster 44 consists of endothelial and perivascular macrophages. (A) Histograms showing relative marker expression for cluster 44 (red) compared to total cells (gray). (B) Imaging mass cytometry of cluster 44 (green) location indicated between nontumor and tumor regions from the same patient. Yellow arrows point toward the location of HLA-DR^+^ CD34^+^ cells (yellow). (C) Waterfall plot of number of VEGFA^+^ and VEGFA^–^ macrophages in proximity to CD34^+^ endothelial cells, 20 μm increments, range 0–100 μm, based on OPAL mIHC from 8 patient with HCC. (D) Number of VEGFA^+^ macrophages in proximity to CD34^+^ endothelial cells compared to nonendothelial cells (CD34^–^ VEGFA^−^), 20 μm increments, range 100 μm, based on OPAL mIHC from 8 patients with HCC. (E) Representative images of OPAL mIHC of HCC tumor tissue stained for HLA-DR (red), FXIIIA (green), VEGFA (cyan), CD34 (magenta), and DAPI (blue) for four out of eight patients with HCC are shown. Scale bar = 50 μm. Two-way ANOVA with the uncorrected Fisher test was used, *p < 0.05, **p < 0.001. Abbreviation: mIHC, multiplex immunohistochemistry.

Having identified cluster 44 at the center of multiple cellular neighborhood interactions, this cell cluster was investigated further. Closer examination of the expression markers associated with cluster 44 revealed expression of both CD45 (indicating a hematopoietic cell) and tumor endothelial marker CD34 (Figure 5B). This prompted us to closely examine all images to determine potential close interactions between 2 different cell types (Supplemental Figure S8, http://links.lww.com/HC9/B51). We found that cluster 44 was, in fact, 2 discrete cells in close proximity: a tumor endothelial cell characterized by CD34 expression and a myeloid cell that expressed CD14, CD16, CD45, CD68, CD86, CD206, FXIIIA, and HLA-DR, but not CD11c (Figure 5A). Based on the proximity of this myeloid cell to the tumor endothelial cell with reference to the phenotypic markers expressed, including FXIIIA that is expressed by macrophages,^51^,^52^,^53^,^54^,^55^ we hypothesized the myeloid cell to be a PVM. Further staining in additional specimens of HCC tissues from 8 patients using mIHC, which allowed for greater image resolution, showed that many of these PVM-like cells also expressed VEGFA, suggesting they are indeed PVMs (Figure 5E, Supplemental Figure S2, http://links.lww.com/HC9/B51).^56^,^57^,^58^ This staining also showed VEGFA^–^ macrophages in close association with CD34^+^ endothelial cells, indicating that both VEGFA^+^ and VEGFA^–^ macrophages preferentially reside near CD34^+^ endothelial cells (Figure 5C). Analysis of spatial interactions of VEGF^+/–^ macrophages to CD34^+^ endothelial cells in HCC tumor tissue from these 8 patients identified that VEGFA^+^ and VEGFA^–^ macrophages preferentially reside 0–20 μm from CD34^+^ endothelial cells (Figure 5C). Although there was no significant difference in the number of VEGFA^+^ macrophages compared to VEGF^–^ macrophages in the proximity of CD34^+^ endothelial cells, significantly more VEGFA^+^ and VEGFA^–^ macrophages reside closely (0–20 μm) to CD34^+^ endothelial cells compared to those residing further away, 40–60, 60–80, and 80–100 μm (Figure 5C). In contrast, no significant difference in the number of VEGFA^+^ macrophages residing close to nonendothelial cells (0–20 μm) was found when compared to those residing further away (20–100 μm, 20 μm increments) (Figure 5D).

**FIGURE 6:**
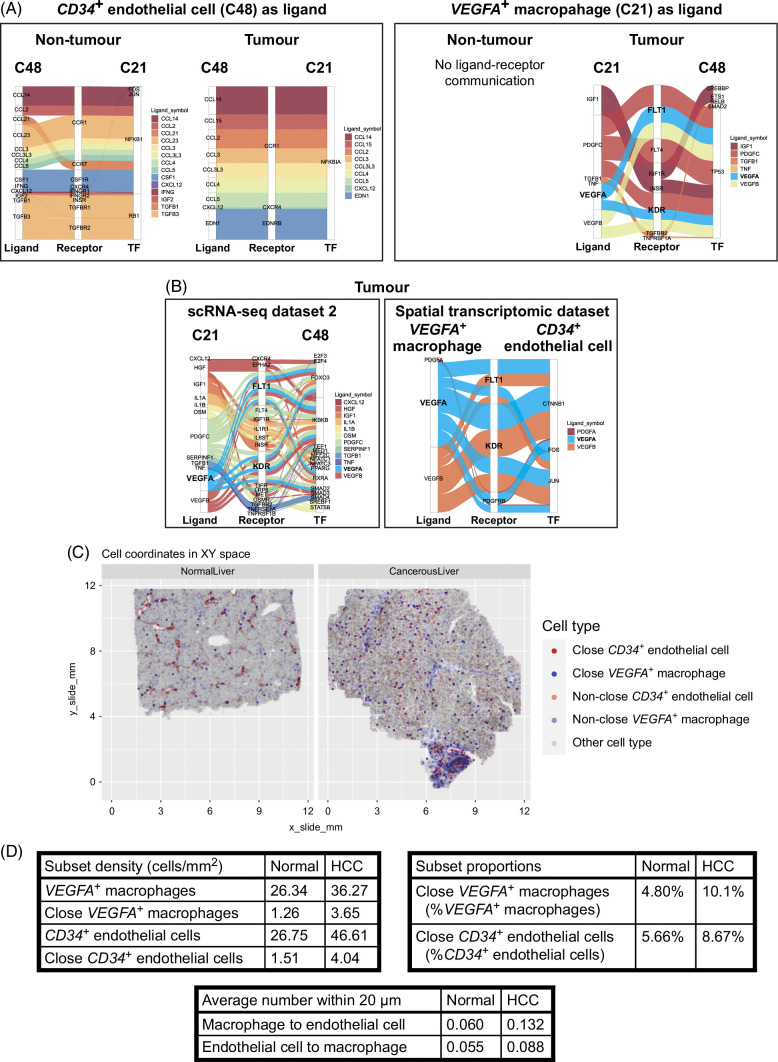
*VEGFA* ^*+*^ macrophages and *CD34* ^*+*^ endothelial cells have a ligand-receptor communication through *VEGFA* within tumors. (A) Ligand-receptor communication between *CD34* ^*+*^ endothelial cells and *VEGFA* ^*+*^ macrophages. Ten patients with HCC and paired nontumor and tumor regions were used. (B) Ligand-receptor communication between *VEGFA* ^*+*^ macrophages (C48, ligand) and *CD34* ^*+*^ endothelial cells (C21, receptor) within the tumor. The scRNA-seq data set consisted of paired nontumor and tumor regions from 6 patients with HCC. The spatial transcriptomic data set consisted of 1 liver section from 1 patient with HCC. *VEGFA* ligand and VEGF receptor genes (*FLT1* and *KDR*) are bolded. (C) Visualization of the spatial transcriptomic data for normal and HCC liver. *CD34* ^*+*^ endothelial cells are red, and *VEGFA* ^*+*^ macrophages are blue. Close cells (within 20 μm) are solid, whereas nonclose cells are translucent. Other cell types are gray. (D) Quantification of relative levels of *CD34* ^*+*^ endothelial cells and *VEGFA* ^*+*^ macrophages in the spatial transcriptomic data set (n = 1). Abbreviation: scRNA-seq, single-cell RNA-sequencing.

Spatial transcriptomics is still a relatively new technology, with most studies using spot-based technologies that quantify regions holding several cells,^59^,^60^,^61^,^62^,^63^ with only a handful that provide single-cell resolution.^64^ To determine whether any cell-cell communication was occurring between the CD34^+^ endothelial cells and the VEGFA^+^ PVM, publicly available single-cell transcriptomic data were analyzed. The GSE149614 data set consisted of tumor resections from newly diagnosed patients with HCC with paired nontumor and tumor tissue cell dissociates.^40^ The preannotated data included endothelial cells expressing *CD34* (C48) and macrophages expressing *VEGFA* (C21) that most resembled PVM. Several algorithms are available to quantify the probability of ligand-receptor communication. These have been comprehensively reviewed in *Nature Reviews Genetics*.^65^ Here, “CellCall” was used,^44^ as it was previously used to effectively investigate myeloid cell-endothelial cell communication through VEGF in patients with colorectal cancer.^66^ When C48 (*CD34* ^*+*^ endothelial cells) acted as the ligand, several chemokine ligands were predicted to interact with chemokine receptors on C21 in both nontumor and tumor regions (Figure 6A). When C21 (*VEGFA* ^*+*^ macrophages) acted as the ligand, there was communication within the tumor but not nontumor tissue with endothelial cell (C48) receptors. Furthermore, *VEGFA* and *TGFB1* were predicted ligands from C21, suggesting the VEGFA^+^ macrophages are communicating with CD34^+^ endothelial cells. To expand upon these results, additional validation was done. Similar findings were observed in 2 additional publicly available cohorts, including a second scRNA-seq cohort and a subcellular spatial transcriptomic data set using CosMx (Figure 6B). This ligand-receptor interaction involved endothelial cell expression of the VEGF receptor genes *FLT1* and *KDR*, which encode VEGF receptors 1 and 2, respectively. *VEGFA* ^*+*^ macrophages within 20 µm of *CD34* ^*+*^ endothelial cells are visualized in the normal and cancerous liver (Figure 6C). The levels of *VEGFA* ^*+*^ macrophages and *CD34* ^*+*^ endothelial cells are summarized in Figure 6D. Despite n = 1, there was a higher density and proportion of *VEGFA* ^*+*^ macrophages and *CD34* ^*+*^ endothelial cells within the cancerous liver compared to the normal liver, and a higher level of cellular neighborhood interactions (Figure 6D). These transcriptomic data reveal the potential for cellular interaction between *VEGFA* ^+^ macrophages and *CD34* ^+^ endothelial cells through *VEGFA* and VEGF receptors 1 and 2, *FLT1*, and *KDR*. Together, the mIHC, scRNA-seq, and spatial transcriptomic data reveal that tumor regions are not only highly enriched with CD34^+^ tumor endothelial cells, but also have VEGFA^+^ PVM that interact with these endothelial cells in that region.

### Perivascular-immune cell aggregates consist of liver-resident CD4^+^ and CD8^+^ T cells, dendritic cells, and macrophages within HCC tumor tissue

**FIGURE 7:**
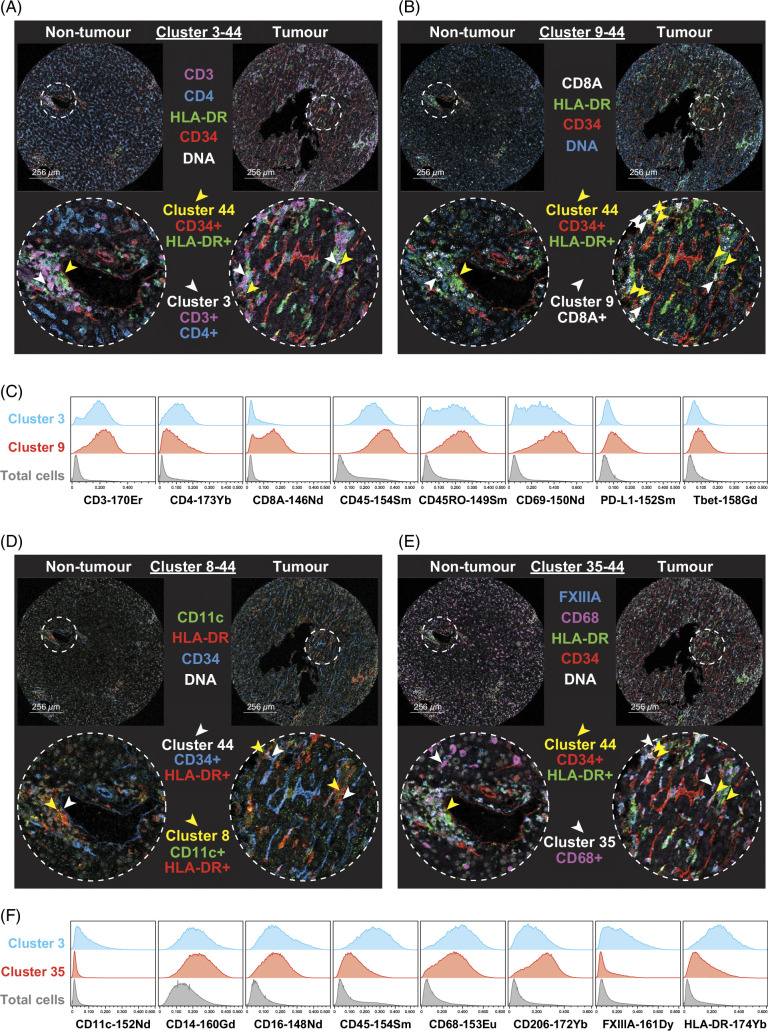
Cluster 44 has a higher number of cellular neighborhood interactions with T cells and myeloid cells within tumor compared to nontumor. (A) Cluster 3 (CD3^+^ CD4^+^ cells, white arrows) and cluster 44 (CD34^+^ HLA-DR^+^ cells, yellow arrows) locations indicated between nontumor and tumor regions. (B) Cluster 9 (CD8A^+^ cells, white arrows) and cluster 44 (CD34^+^ HLA-DR^+^ cells, yellow arrows) locations indicated between nontumor and tumor regions. (C) Histograms representing marker expression of cluster 3 (blue), cluster 9 (red), and total cells (gray). (D) Cluster 8 (CD11c^+^ HLA-DR^+^, yellow arrows) and cluster 44 (CD34^+^ HLA-DR^+^, white arrows) locations indicated between nontumor and tumor regions. (E) Cluster 35 (CD68^+^, white arrows) and cluster 44 (CD34^+^ HLA-DR^+^, yellow arrows) locations indicated between nontumor and tumor regions. (F) Histograms representing marker expression of cluster 8 (blue), cluster 35 (red), and total cells (gray). Representative nontumor and tumor regions are from the same patient. Abbreviation: PD-L1, programmed cell death ligand 1.

Having identified multiple clusters neighboring with cluster 44, we then defined all clusters that were found in close proximity to cluster 44. Clusters 3 (Figure 7A) and 9 (Figure 7B) were T cells (CD45^+^ CD3^+^). Further phenotyping showed cluster 3 to be CD4^+^ CD45RO^+^ CD69^+^, suggestive of liver-resident memory CD4^+^ T cells, and cluster 9 to be CD8A^+^ CD45RO^+^ CD69^+^ Tbet^+^ PD-L1^+^, likely liver-resident memory CD8^+^ T cells (Figure 7C). Both clusters had a higher interaction with cluster 44 within the tumor, suggesting an interaction between CD4^+^ and CD8^+^ T cells with PVMs and tumor endothelium.

Clusters 8 (Figure 7D) and 35 (Figure 7E) were CD45^+^ CD14^+^ HLA-DR^+^ myeloid cells. Cluster 8 was also CD11c^+^ CD16^+^ CD68^+^ CD206^+^ FXIIIA^+^, suggestive of a potential macrophage, while cluster 35 was CD11c^–^ CD16^+^ CD68^+^ CD206^+^ FXIIIA^–^, possibly representing a dendritic cell population (Figure 7F). The increased cellular neighborhood interaction of these cells with PVM-endothelial cell cluster (cluster 44) in the HCC tumor tissue points to an interaction between the tumor endothelium/PVM niche and other myeloid cells within the TME.

**FIGURE 8:**
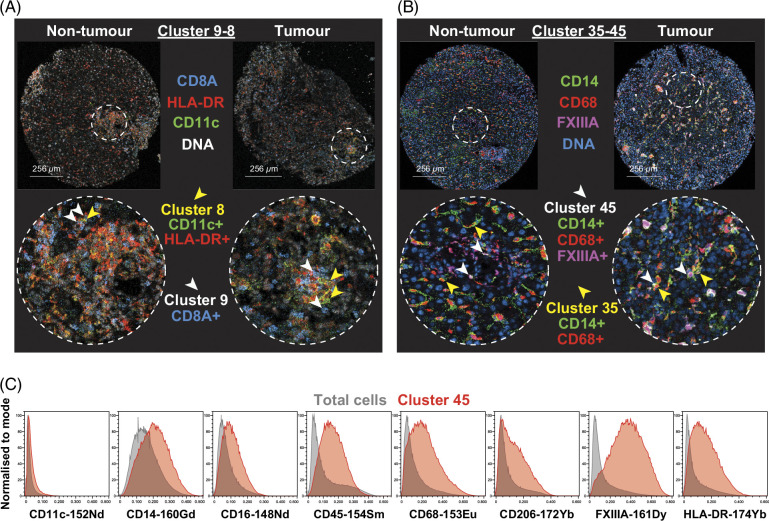
Cellular neighborhood interactions between clusters 8 and 9 and clusters 35 and 45 are higher in tumor than nontumor. (A) Cluster 8 (CD11c^+^ HLA-DR^+^, yellow arrows) and cluster 9 (CD8A^+^, white arrows) locations indicated between nontumor and tumor regions. (B) Cluster 35 (CD14^+^ CD68^+^, yellow arrows) and cluster 45 (CD14^+^ CD68^+^ FXIIIA^+^, white arrows) locations indicated between nontumor and tumor regions. (C) Histograms representing marker expression of cluster 45 (red) and total cells (gray). Representative nontumor and tumor regions are from the same patient.

Cluster 8, a macrophage subset, was also found to have a cellular neighborhood interaction with cluster 9, a memory CD8^+^ T-cell subset (Figure 8A, Supplemental Figure S9, http://links.lww.com/HC9/B51). Clusters 35 (a dendritic cell subset) and 45 were both CD45^+^ CD14^+^ HLA-DR^+^. Cluster 45 had a macrophage phenotype as CD16^+^ CD68^+^ FXIIIA^+^ (Figure 8C). The cellular neighborhood interactions between these 2 myeloid cell populations were also higher in the tumor compared to nontumor regions (Figure 8B) despite the density of cluster 35 being lower in the tumor compared to the nontumor regions (Figure 4E). Together, these single-cell maps reveal that unique intratumoral immune-rich aggregates (consisting of T cells, dendritic cells, and macrophages) interact with PVM and may exert potential protumoral and angiogenic functions. It should be noted that both a CD8^+^ T cell (cluster 9) and a myeloid cell (cluster 8) within the perivascular-immune neighborhood described here express PD-L1. This suggests there are multiple potential cellular targets for atezolizumab (anti-PD-L1) and bevacizumab (VEGF-inhibitor) therapy. These findings are summarized in Supplemental Figure S11, http://links.lww.com/HC9/B51.

Clinical associations were assessed by correlating tumor cellular neighborhood interactions with prognostic and clinicopathological data. There were no associations between tumor cellular neighborhood interactions and cirrhosis, early recurrence (<2 y), microvascular invasion, or viral-associated HCC (Supplemental Figure S10, http://links.lww.com/HC9/B51).

## DISCUSSION

The treatment of HCC remains a major challenge and novel therapeutic strategies are urgently required. Irrespective of its etiology, HCC mostly develops in diseased liver with concomitant chronic inflammation, thus implicating the immune system in both the pathogenesis of the disease as well as tumor control. Understanding the complex interactions between immune cells and the spatial architecture of the TME is, therefore, paramount.^9^,^67^ We used high-dimensional spatial analysis to map multiple cellular neighborhood interactions across 3 regions: the nontumor, invasive margin, and tumor regions. Mapping the immune landscape across these regions showed that only innate cell densities, but not T-cell or B-cell densities, are increased within tumor regions compared to nontumor regions, and that macrophages (likely monocyte-derived) are enriched within tumor tissue. We further identified immune aggregates around perivascular spaces that are unique to the tumor region. Detailed analysis of this region shows that PVMs are at the center of these interactions and are closely associated with not only the tumor endothelial cells, but also multiple immune populations, including liver-resident CD4^+^ and CD8^+^ T cells, macrophages, and dendritic cells. Functionally, we show that these PVMs were associated with VEGFA, which is known to be a key driver of angiogenesis and hypervascularity within tumors. Mining publicly available scRNA-seq and spatial transcriptomic data sets capable of single-cell resolution to interrogate potential ligand-receptor interactions between PVMs and endothelial cells confirms VEGFA-VEGF receptor interactions and suggests that other potential protumoral mechanisms involving TGF-β are active within these unique immune aggregates.

How the TME and its composition are impacted by hepatocarcinogenesis remains unclear. Our data show the enrichment of phenotypically diverse populations of macrophages within the tumor compared to nontumor regions. Based on CD14 expression, these populations were most likely monocyte-derived.^68^,^69^ This is in line with the consensus in the field that tumor-associated macrophages are derived primarily from circulating blood monocytes,^70^ and in human HCC, tumor-associated macrophages arise from CCR2^+^ monocytes.^71^ Interestingly, we found other CD14^–^ macrophage populations were either reduced within tumors or did not show any difference compared to adjacent nontumor tissue. This, together with no changes in CD8^+^ T cell or CD4^+^ T-cell subset densities between tumor and nontumor regions, suggests that the TME may be skewed by the recruitment of monocytes. Furthermore, our findings on several myeloid cell populations regarding their overall numbers and cell-cell interactions should validate and enhance the findings of Wu et al, who devised a myeloid response score using 244 resected human HCC samples.^72^ Tumors with a high myeloid response score were enriched in CD11b^+^, CD15^+^, CD163^+^, CD33^+^, CD68^+^, and CD204^+^ cells. These were thought to be immunosuppressive in nature and associated with poor prognosis. In addition, Kurebayashi et al found that macrophages were the most common immune cell detected in 919 regions from 158 resected HCC tumors.^73^ CD68 is commonly used as a liver tumor-associated macrophage marker,^74^ although it is expressed on multiple cells in the liver and HCC tissue, and this subset was found to be increased in our tumors. Similarly, CD206^+^ CD14^+^ monocyte-derived macrophages were also enriched within the tumor in our study. A dysregulated balance toward CD206^+^ M2 polarized macrophages has also been associated with aggressive tumor phenotypes and worse prognosis in HCC.^75^

Where these macrophages localize within tumor tissue is critical to our understanding of how the immune environment impacts tumor development, persistence, as well as tumor control. Many previous studies have interrogated the TME in HCC using various techniques, including mIHC,^72^,^73^,^76^ IMC,^22^,^23^,^24^,^27^ and multimodal genomic analysis incorporating scRNA-seq and TCR receptor sequencing.^77^,^78^,^79^ In fact, all 4 studies that have used IMC to study the immune cell landscape have observed close interactions between T cell and macrophage subsets, which has been associated with HCC etiologies,^27^ response to immunotherapies,^22^,^23^ and overall survival.^24^ CCR2^+^ macrophages have been found near CD31^+^ endothelial cells in HCC tumors.^80^ *FOLR2* ^+^ macrophages and *PLVAP* ^+^ endothelial cells have also been identified as part of an immunosuppressive oncofetal neighborhood,^20^ which are associated with relapse and response to immunotherapy.^81^ *VEGFA* from cancer cells was identified as a core ligand that regulates this oncofetal neighborhood.^20^,^81^ Our study builds upon these works by specifically examining interactions within only the tumor microenvironment and by providing a quantitative spatial analysis of protein expression at a subcellular level. We not only identified a unique immune cell neighborhood within HCC tumors, but also an association with tumor vasculature. Through this spatial mapping we have identified a cellular neighborhood around the endothelium and VEGFA^+^ PVMs that may be of importance, particularly as nonresponders of combination cabozantinib (a tyrosine kinase inhibitor that targets VEGF receptor) and nivolumab (anti-programmed cell death 1) have a higher interaction of T cells and Arginase 1^hi^ macrophages compared to responders.^22^,^23^ In fact, the identified perivascular-immune neighborhood has multiple cells that are potential therapeutic targets of combination atezolizumab-bevacizumab, expressing PD-L1 or VEGF.

The close physical association between tumor endothelial cells and macrophages prompted us to define them as PVMs. Currently, there is no consensus on the definition of PVMs as they can have different phenotypes across tissues. However, a unifying feature is that they lie near blood vessels, either in direct contact or located within 1 cell thickness from it.^58^ However, PVMs have not yet been described in healthy adult liver tissue. In diseased tissues such as cancer, these cells have been shown to express VEGFA.^57^,^58^ We also show that these PVMs are indeed able to produce VEGFA. Importantly, preclinical models show that PVMs are also largely derived from circulating monocytes.^57^ In line with this, Matsubara et al found that the majority of angiopoietin (tyrosine kinase with immunoglobulin and epidermal growth factor homology domains 2^+^) expressing macrophages in liver cancer was located in the perivascular areas of tumor tissue, and their presence not only correlated with the degree of angiogenesis, but also with the levels of their counterparts in blood.^82^ Several other functions of PVMs have been described both in other healthy adult tissues (phagocytosis, antigen presentation, and immune regulation) and in the progression of pathological conditions (angiogenesis, metastasis, immunosuppression, recruitment of other leukocytes, and posttherapy relapse).^58^ It is tempting to postulate that PVMs are critical not only for angiogenesis and hypervascularity in HCC through the secretion of VEGFA, but are also responsible for the “leaky” vessels seen in HCC that compromise immune control mechanisms. There is evidence from preclinical models to support both these possibilities. Tyrosine kinase with immunoglobulin and epidermal growth factor homology domains 2-expressing monocytes were found to be critical for tumor angiogenesis,^83^ supporting the fact that monocyte-derived cells within tumors are key for angiogenesis. In addition, in a murine model of breast cancer, macrophage-specific depletion of VEGFA reduced both vascular permeability and circulating tumor cells while restoring vascular junctions.^56^

Our tissue microarray selection criteria of highly abundant tumor-infiltrating lymphocytes (TILs) on hematoxylin and eosin suggest we have selected areas that share features of the *Inflamed Class* of HCC.^79^,^84^ This class represents <35% of HCC tumors and is characterized by a microenvironment with increased interferon signaling and higher immune infiltration (increased TILs, macrophages, tertiary lymphoid structures, and elevated expression of checkpoint molecules) compared with noninflamed tumors.^79^ The inflamed subclass exhibits a significantly higher fraction of CD8^+^ T cells and M1 macrophages when compared to the noninflamed profiles. However, despite the presence of high TILs, these tumors persist. There could be many factors that contribute to this tumor persistence, including enrichment of clonally exhausted CD8^+^ T cells and regulatory T cells within HCC tissues.^77^ Our finding of no difference in the T-cell density between tumor and nontumor tissues is hence of interest. We also found that CD4^+^ T cells were more abundant than CD8^+^ T cells within the HCC TME, which is consistent with previous studies.^73^ Furthermore, CD4^+^ T cells expressing CD69 were the most common subset of CD4^+^ T cells across all regions in our study. Importantly, we found close interactions between these T cells and PVMs in the perivascular space. This niche could facilitate regulatory interactions for effector and resident T cells. We also identified multiple myeloid cells interacting with T cells within this niche, although the functional role of these myeloid cells remains unknown. Further studies are required to identify how and why T cells are potentially impaired within such tumor regions.

The findings from our study have several clinical implications. The identification of increased TIL density within a perivascular-immune neighborhood (consisting of tumor endothelial cells and PVMs) warrants a discussion of current therapeutic approaches that target immune-vascular cross-talk and the role of vascular normalization in HCC.^85^ The tumor vasculature in HCC is structurally and functionally abnormal, and the tumor vessels are excessively leaky.^86^ Immune-vascular cross-talk underpins the rationale for using bevacizumab, an anti-VEGFA monoclonal antibody that normalizes tumor vasculature, in conjunction with immune checkpoint inhibitors in advanced unresectable HCC.^5^ Patients with advanced colorectal cancer receiving combined FOLFOX and bevacizumab have decreased VEGFB-mediated angiogenesis, including reduced myeloid cell-endothelial cell communication through VEGF as identified by scRNA-seq.^66^ In preclinical HCC models, antiangiogenic therapy in conjunction with anti-programmed cell death 1 therapy further promotes vascular normalization mediated by CD4^+^ T cells.^17^ Recently, biomarkers for response to systemic therapies in advanced HCC have been developed. Haber and colleagues found that tumors expressing high amounts of an 11-gene signature (IFNAP) captured a unique immune microenvironment characterized by a high gene expression of activated CD4^+^ memory T cells, M1 macrophages, and plasma cells. This novel signature predicted response and survival in patients treated in the first-line setting with anti-programmed cell death 1 monotherapy.^87^ However, this study did not incorporate spatial analyses of the tissue to determine the precise location and immune cell interactions within the immune microenvironment captured by the IFNAP gene signature.

There are notable limitations to our study. Single-cell sequencing of TILs in HCC has identified 11 unique subsets: 5 CD8^+^ and 6 CD4^+^ T-cell clusters, including naïve, effector, and exhausted T cells and mucosal-associated invariant T cells.^77^ We were unable to identify some of these subsets (undefined lymphocytes and undefined nonlymphocyte immune cells) due to constraints on the number of markers that can be included in a single IMC panel and limitations of available effective antibodies to identify some cell types. Cell segmentation is a known limitation when defining single cells sectioned from a 3-dimensional organ. As such, cluster 44 was first identified as a single cell rather than 2 discrete closely interacting cells. This issue was resolved by examining representative images to confirm the presence of 2 discrete cells. Functionality of the PVM population was inferred by in silico analysis, rather than cell isolation and functional culture experiments ex vivo. Finally, our sample size was small. Correlations with prognostic and clinicopathological data were performed, but no associations were found, perhaps related to the limited sample size.

In summary, our study has identified a novel myeloid population in HCC characteristic of PVMs and identified several immune cell and vascular neighborhood interactions occurring more commonly within HCC tumors. Using IMC, we were able to comprehensively immunophenotype and spatially map the immune landscape of human HCC tumor and adjacent nontumor tissue. Further studies are required to evaluate the function of these immune cells, their effect on immune-mediated tumor control and therapeutic responses, and their role in hepatocarcinogenesis and HCC progression.

## Supplementary Material
