# Supplementary material for: Spatial mapping of the HCC landscape identifies unique intratumoral perivascular-immune neighborhoods
Source: Hepatol Commun. 2024 Oct 17;8(11):e0540. doi: 10.1097/HC9.0000000000000540 (PMC11495755; doi:10.1097/HC9.0000000000000540)
Supplement: Supplementary file 1 [file hc9-8-e0540-s001.pdf]

Spatial mapping of the hepatocellular carcinoma landscape identifies unique intratumoural perivascular-immune neighbourhoods

Felix Marsh-Wakefield[†1,2], Cositha Santhakumar[†1,2,3], Angela L. Ferguson[†1,2], Thomas M. Ashhurst[4,5], Joo-Shik Shin[6,7], Fiona H. X. Guan[3], Nicholas J. Shields[4], Barry J. Platt[2], Givanna H. Putri[8], Ruta Gupta[6,7], Michael Crawford[9], Carlo Pulitano[9,10], Charbel Sandroussi[9,10], Jerome M. Laurence[9,10], Ken Liu[1,3,4], Geoffrey W. McCaughan[‡1,3,4], Umaimainthan Palendira[‡2]

[†/‡]Authors contributed equally

[1]Liver Injury & Cancer Program, Centenary Institute

[2]Human Immunology Laboratory, School of Medical Sciences, Faculty of Medicine and Health, The University of Sydney

[3]A.W. Morrow Gastroenterology and Liver Centre, Royal Prince Alfred Hospital

[4]School of Medical Sciences, Faculty of Medicine and Health, The University of Sydney

[5]Sydney Cytometry Core Research Facility, The University of Sydney

[6]Central Clinical School, Sydney Medical School, The University of Sydney

[7]Department of Tissue Pathology and Diagnostic Oncology, Royal Prince Alfred Hospital, NSW Health Pathology

[8]The Walter and Eliza Hall Institute of Medical Research and The Department of Medical Biology, The University of Melbourne, Australia

[9]Australian National Liver Transplant Unit, Royal Prince Alfred Hospital

[10]Royal Prince Alfred Institute of Academic Surgery, University of Sydney

Table of contents

Supplementary Figure 1 – Single marker IMC staining 3-7

Supplementary Figure 2 – Single marker OPAL mIHC 8

Supplementary Figure 3 – Manual gating strategy of immune cell subsets 9

Supplementary Figure 4 – Clustering of single cells 10-11

Supplementary Figure 5 – scANVI cluster annotation transfer 12

Supplementary Figure 6 – Phenotypically similar clusters were combined 13

Supplementary Figure 7 – T cell and myeloid cell subset levels across regions 14-15

Supplementary Figure 8 – Representative images of parameters that contributed to differences between non-tumour and tumour regions 16-22

Supplementary Figure 9 – T cell marker expression 23-24

Supplementary Figure 10 – No association between clinical parameters and tumour neighbourhood interaction 25

Supplementary Figure 11 – Summary figure 26

Supplementary Table 1 – Patient cohort 27

Supplementary Table 2 – Imaging mass cytometry antibody panel 28-29

Materials and methods 30-35

Complete reference list 36-40

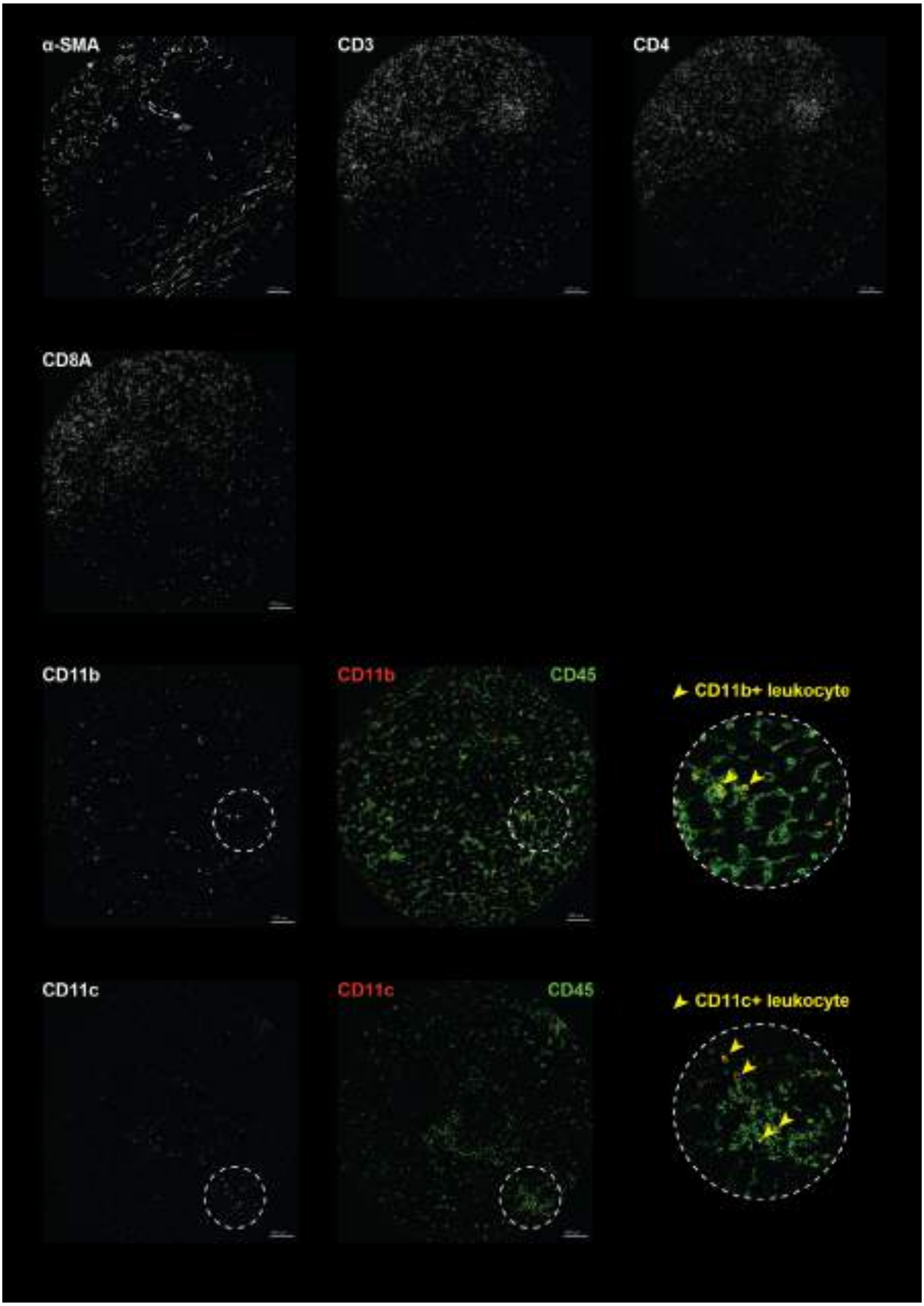
α-SMA
CD3
CD4
CD8A
CD11b
CD11b
CD45
CD11b+ leukocyte
CD11c
CD11c
CD45
CD11c+ leukocyte

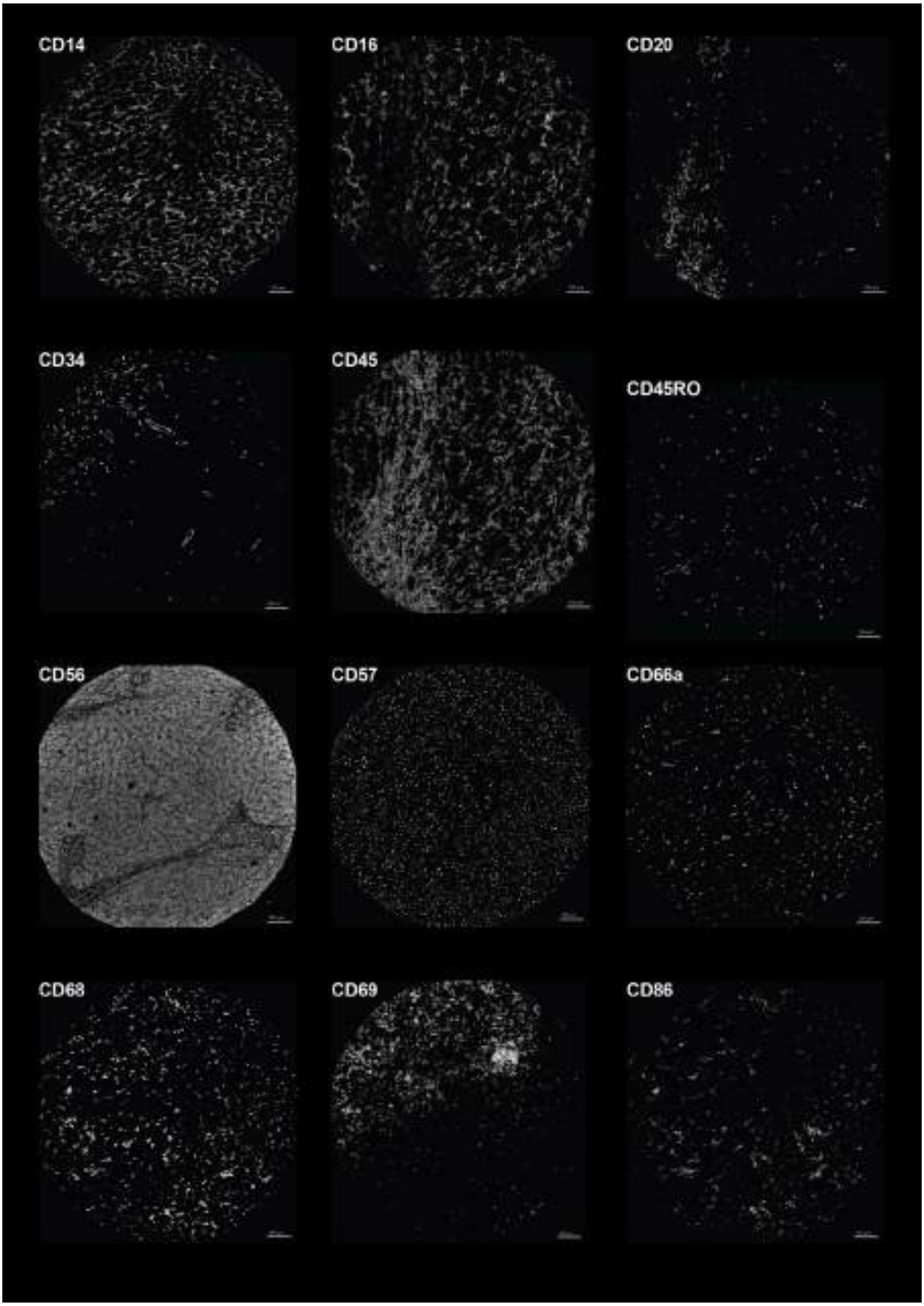
CD14
CD16
CD20
CD34
CD45
CD45RO
CD56
CD57
CD66a
CD68
CD69
CD86

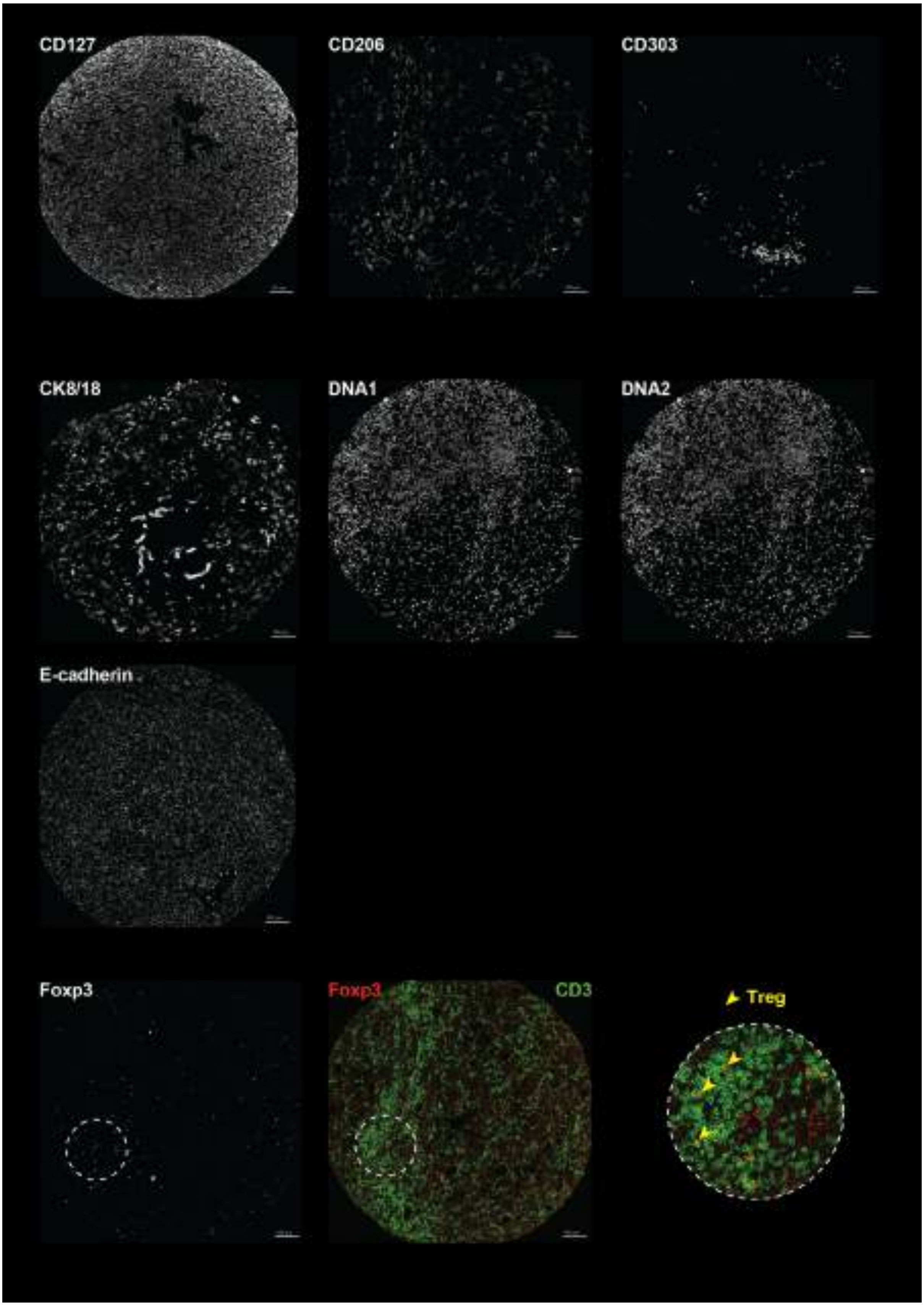
CD127
CD206
CD303
CK8/18
DNA1
DNA2
E-cadherin
Foxp3
Foxp3
CD3
Treg

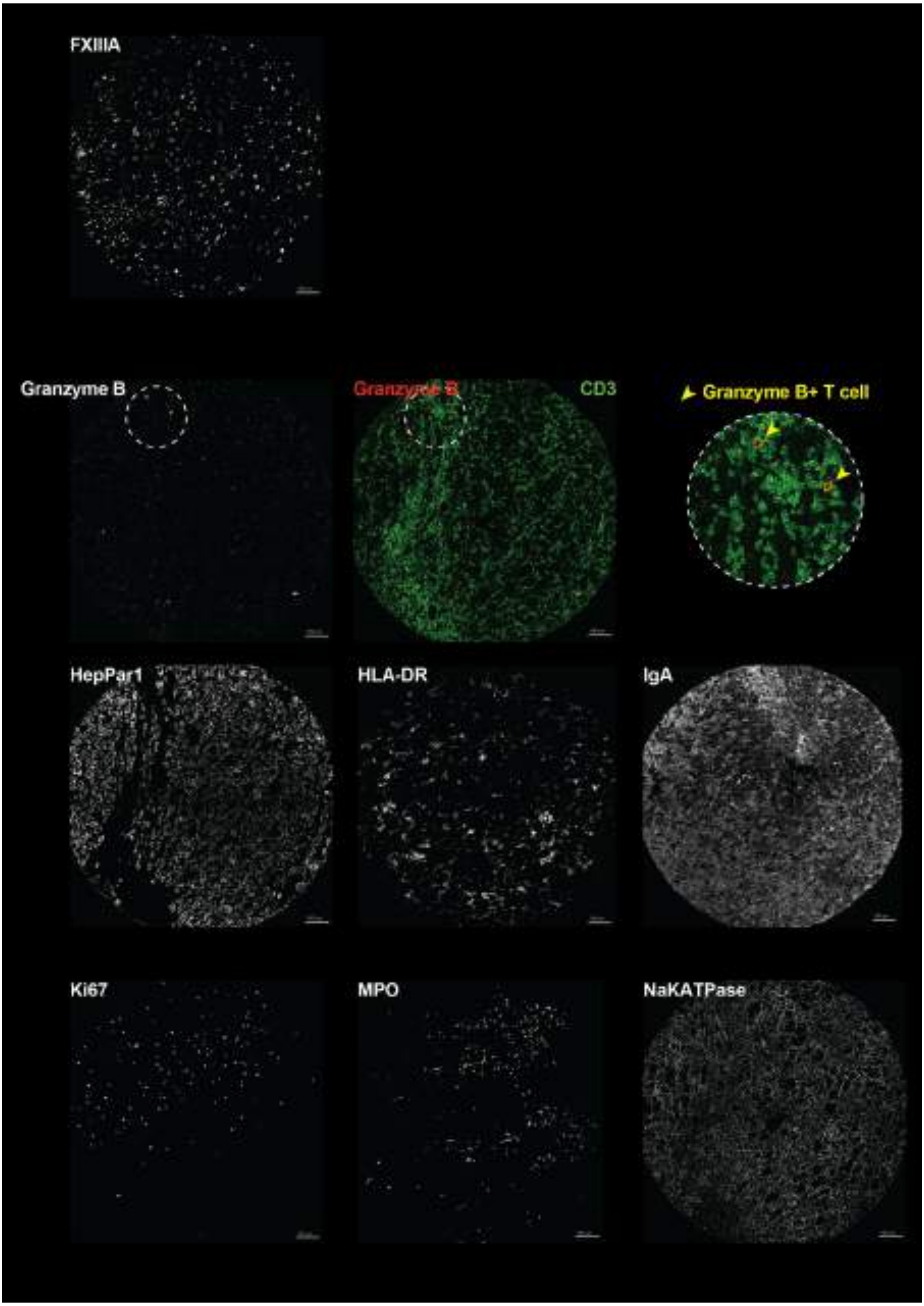
FXIIIA
Granzyme B
Granzyme B
CD3
Granzyme B+ T cell
HepPar1
HLA-DR
IgA
Ki67
MPO
NaKATPase

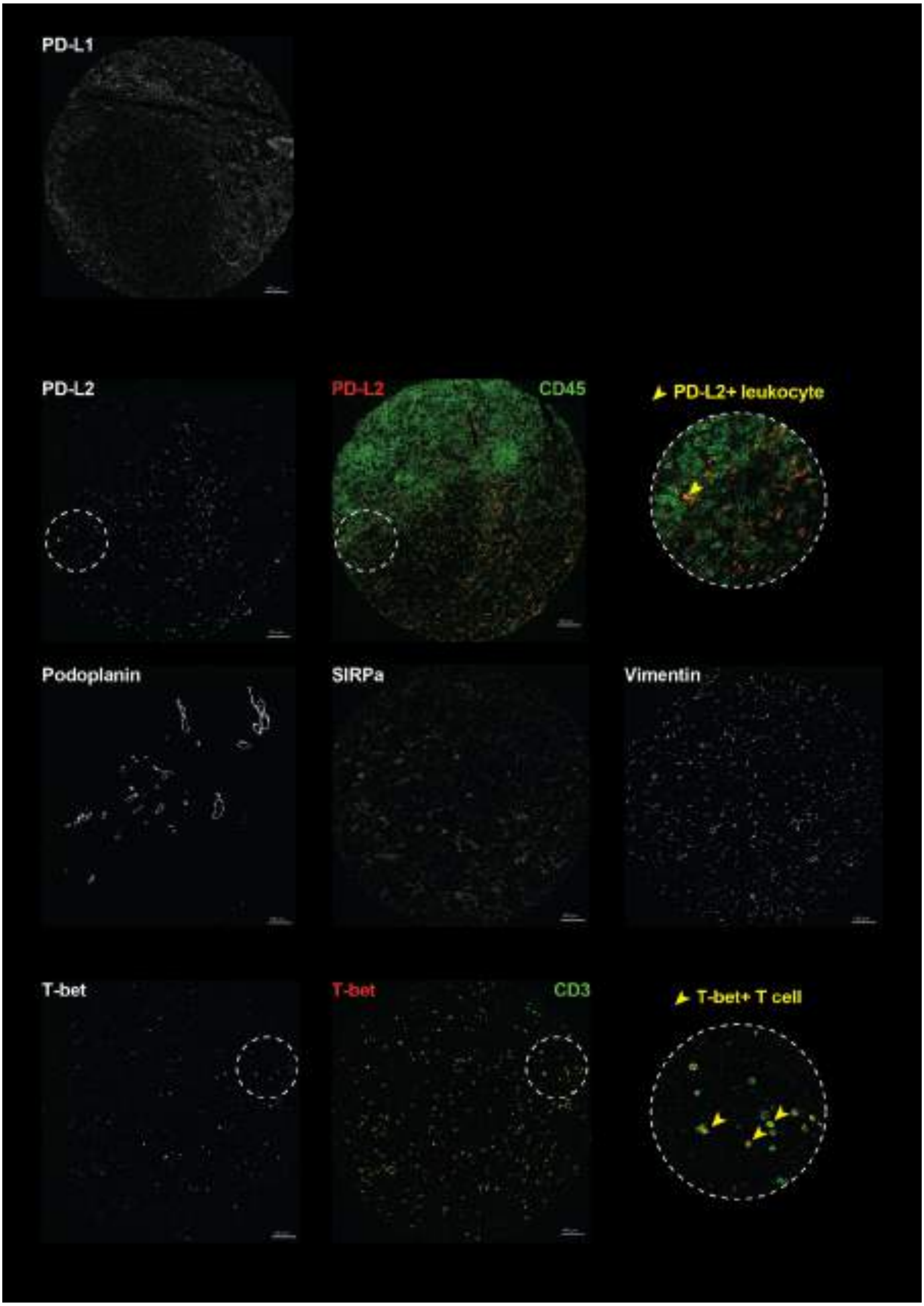

Supplementary Figure 1 – Single marker IMC staining. Representative images for individual IMC antibodies.

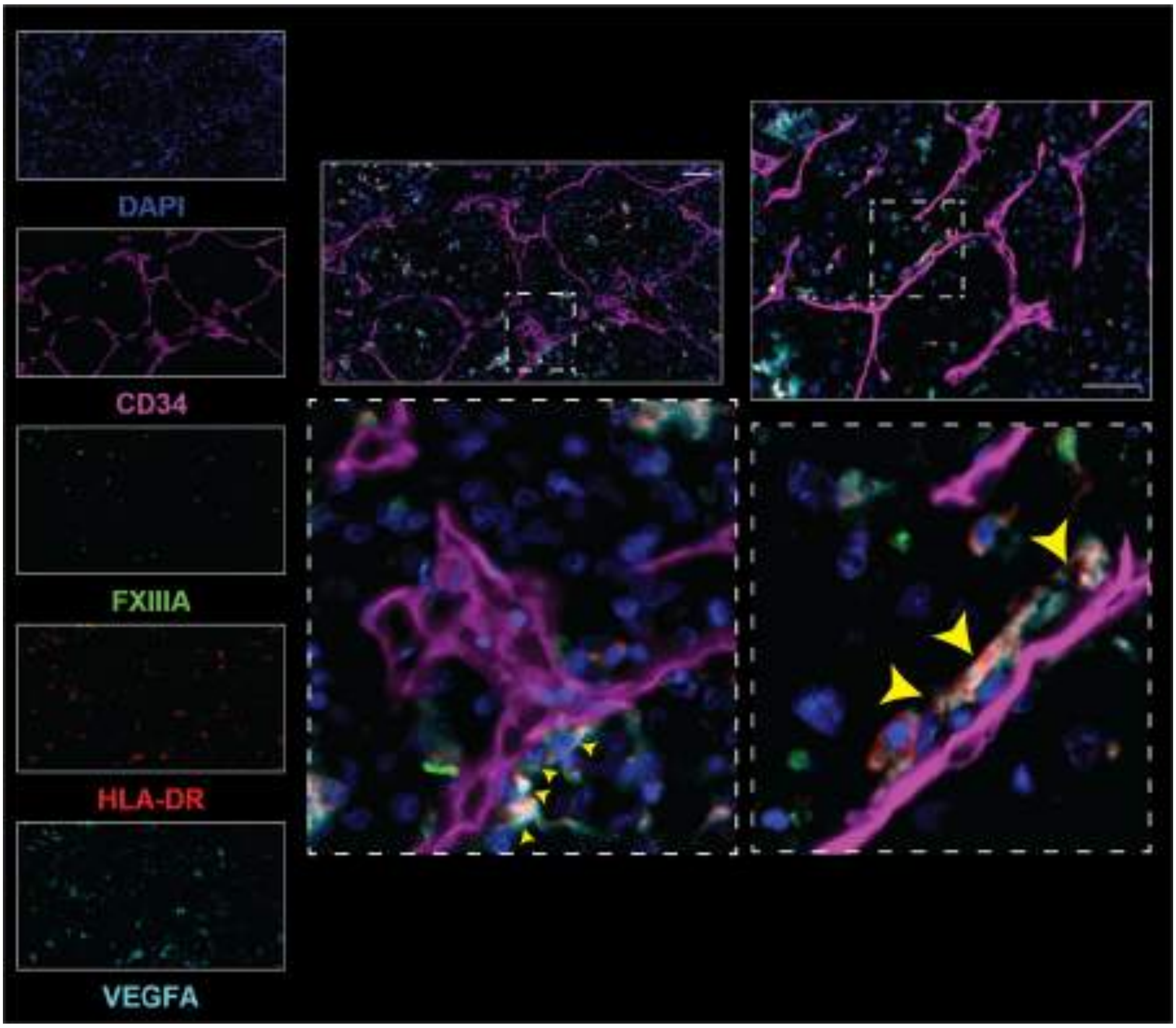

Supplementary Figure 2 – Single marker OPAL mIHC. Representative images for individual OPAL mIHC antibodies. Yellow arrows indicate $VEGFA^+$ macrophages.

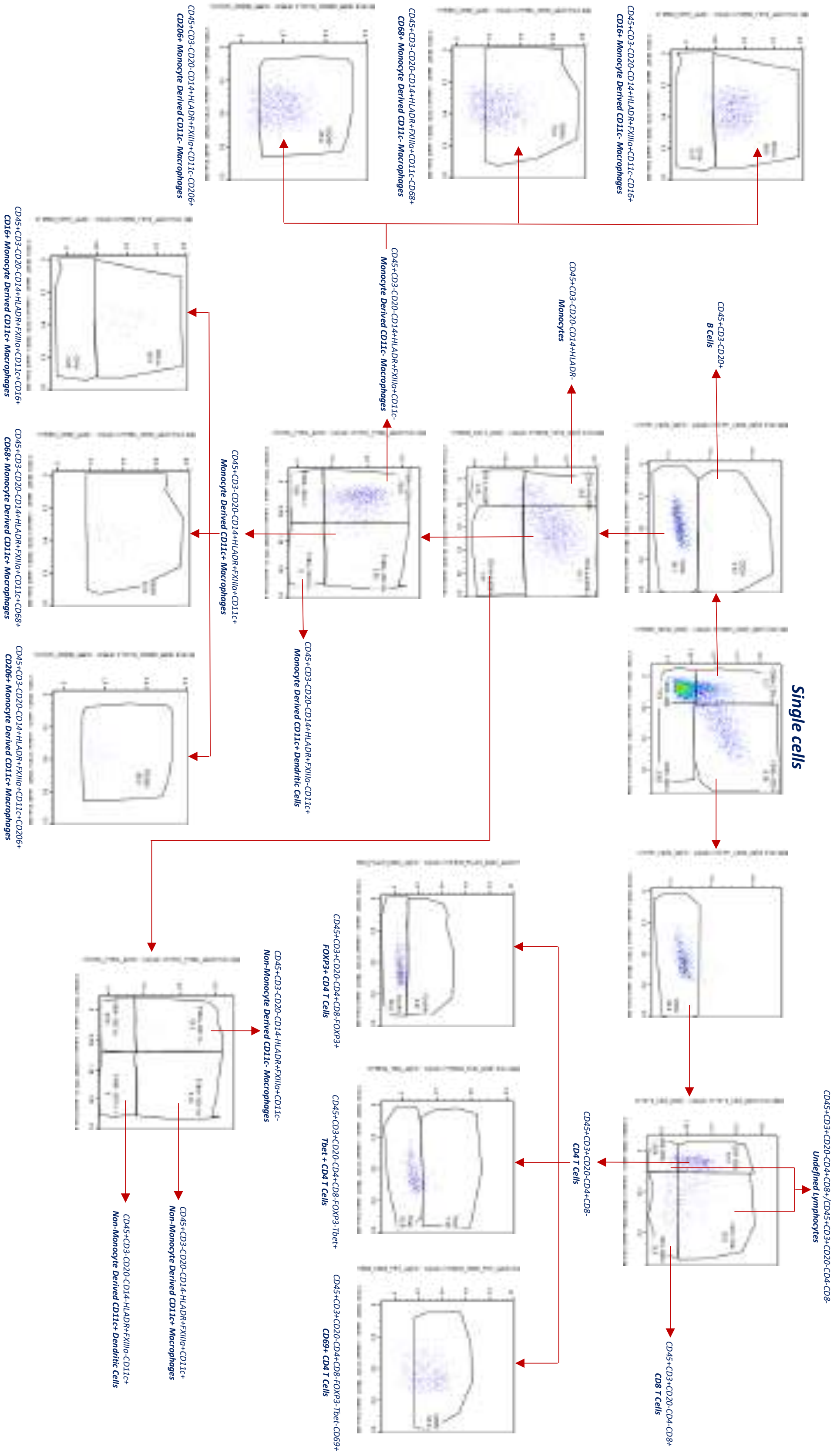

Supplementary Figure 3 – Manual gating strategy of immune cell subsets. Representative gating strategy identifying immune cell subsets.

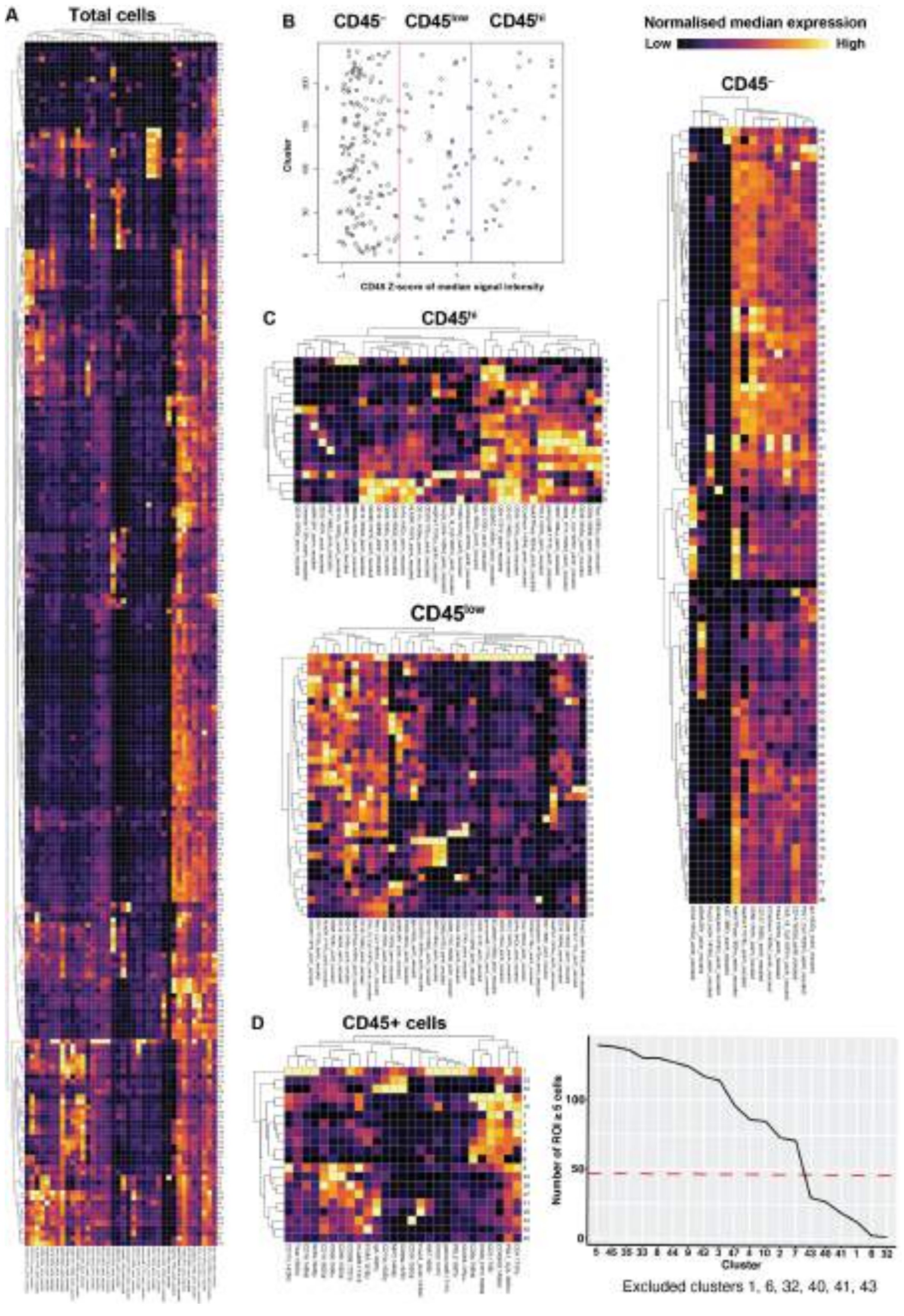
A
Total cells
B
CD45⁻
CD45low
CD45hi
Normalised median expression
Low
High
Cluster
CD45 Z-score of median signal intensity
C
CD45hi
CD45low
CD45⁻
D
CD45+ cells
Number of ROI ≥ 5 cells
Cluster
Excluded clusters 1, 6, 32, 40, 41, 43

Supplementary Figure 4 – Clustering of single cells. A) Total cells were clustered using X-shift. B) The median signal intensity of CD45 was calculated for each cluster before running z-score normalisation. Red vertical line is at zero, whereby a negative cluster indicates no CD45 expression, whilst a positive value indicates CD45 expression. The blue line represents the absolute value of the smallest value, used to differentiate between $CD45^{low}$ and $CD45^{hi}$ populations. C) Cells then underwent X-shift clustering within each of the three groups ($CD45^{-}$, $CD45^{low}$, $CD45^{hi}$) resulting in 91, 36, and 18 clusters respectively. Similar clusters were then combined using the function simprof as part of the ‘clustsig’ R package, generating 47 clusters (20 $CD45^{+}$ clusters and 27 $CD45^{-}$ clusters). D) The number of $CD45^{+}$ clusters with ≥5 cells in each region were calculated. Clusters with <5 cells across less than a third of total regions were excluded, which removed six clusters. Heatmaps show the scaled median signal expression of each marker (columns) across each cluster (rows).

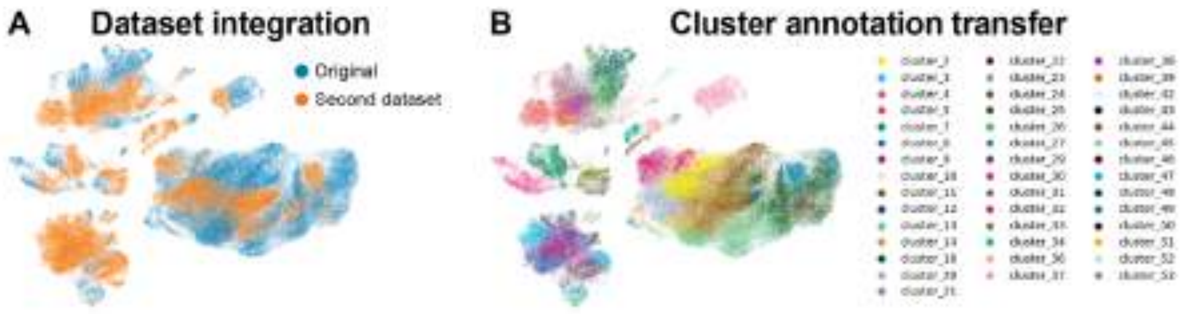

Supplementary Figure 5 – scANVI cluster annotation transfer. Cluster annotations were transferred from initial single-cell RNA-sequencing (scRNA-seq) dataset (GSE149614) to a second scRNA-seq dataset (https://data.mendeley.com/datasets/skrx2fz79n/1). A) UMAP visualising the integrated original reference dataset (orange) with the second dataset (blue). B) UMAP of predicted annotated clusters.

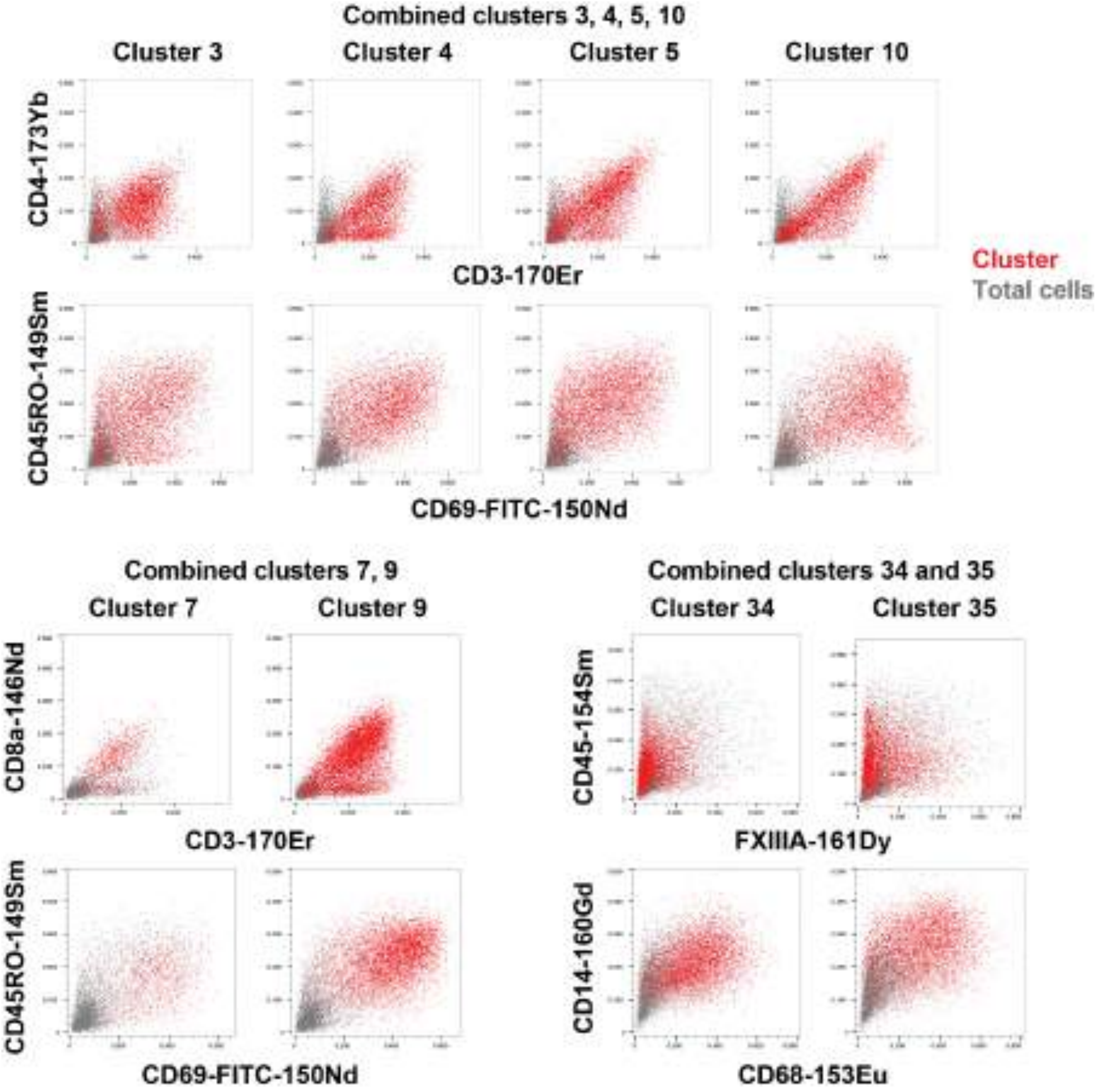

Supplementary Figure 6 – Phenotypically similar clusters were combined. $CD45^+$ clusters that were phenotypically similar were combined. Representative images show each cluster (red) compared to total cells (grey).

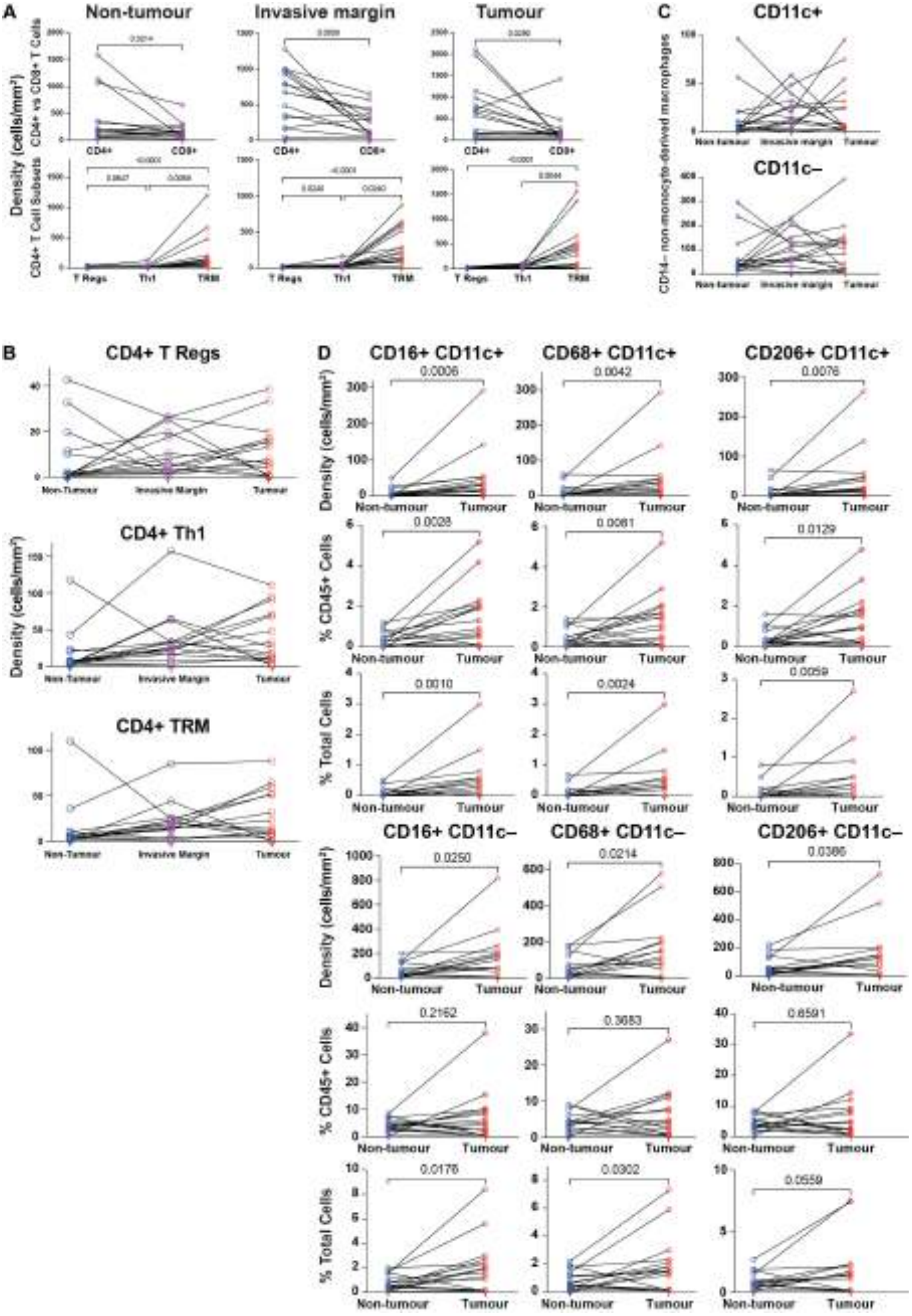
A
Non-tumour
Invasive margin
Tumour
Density (cells/mm²)
CD4+ vs CD8+ T Cells
CD4+ T Cell Subsets
CD4+
CD8+
T Regs
Th1
TRM
C
CD11c+
CD11c–
CD14– non-monocyte-derived macrophages
Non-tumour
Invasive margin
Tumour
B
CD4+ T Regs
CD4+ Th1
CD4+ TRM
Density (cells/mm²)
Non-Tumour
Invasive Margin
Tumour
D
CD16+ CD11c+
CD68+ CD11c+
CD206+ CD11c+
0.0006
0.0042
0.0076
0.0028
0.0081
0.0129
0.0010
0.0024
0.0059
CD16+ CD11c–
CD68+ CD11c–
CD206+ CD11c–
0.0250
0.0214
0.0386
0.2162
0.3683
0.6591
0.0176
0.0302
0.0559
Density (cells/mm²)
% CD45+ Cells
% Total Cells
Non-tumour
Tumour

Supplementary Figure 7 – T cell and myeloid cell subset levels across regions. A) Densities of T cell subsets across regions. B) Densities of $CD4^+$ T cell subsets. Friedman test with Dunn's multiple comparison corrections was done. C) Densities of $CD14^-$ non-monocyte-derived macrophages across regions. D) Density and proportions (% of $CD45^+$ cells or total cells) across myeloid cell subsets. Wilcoxon test was used.

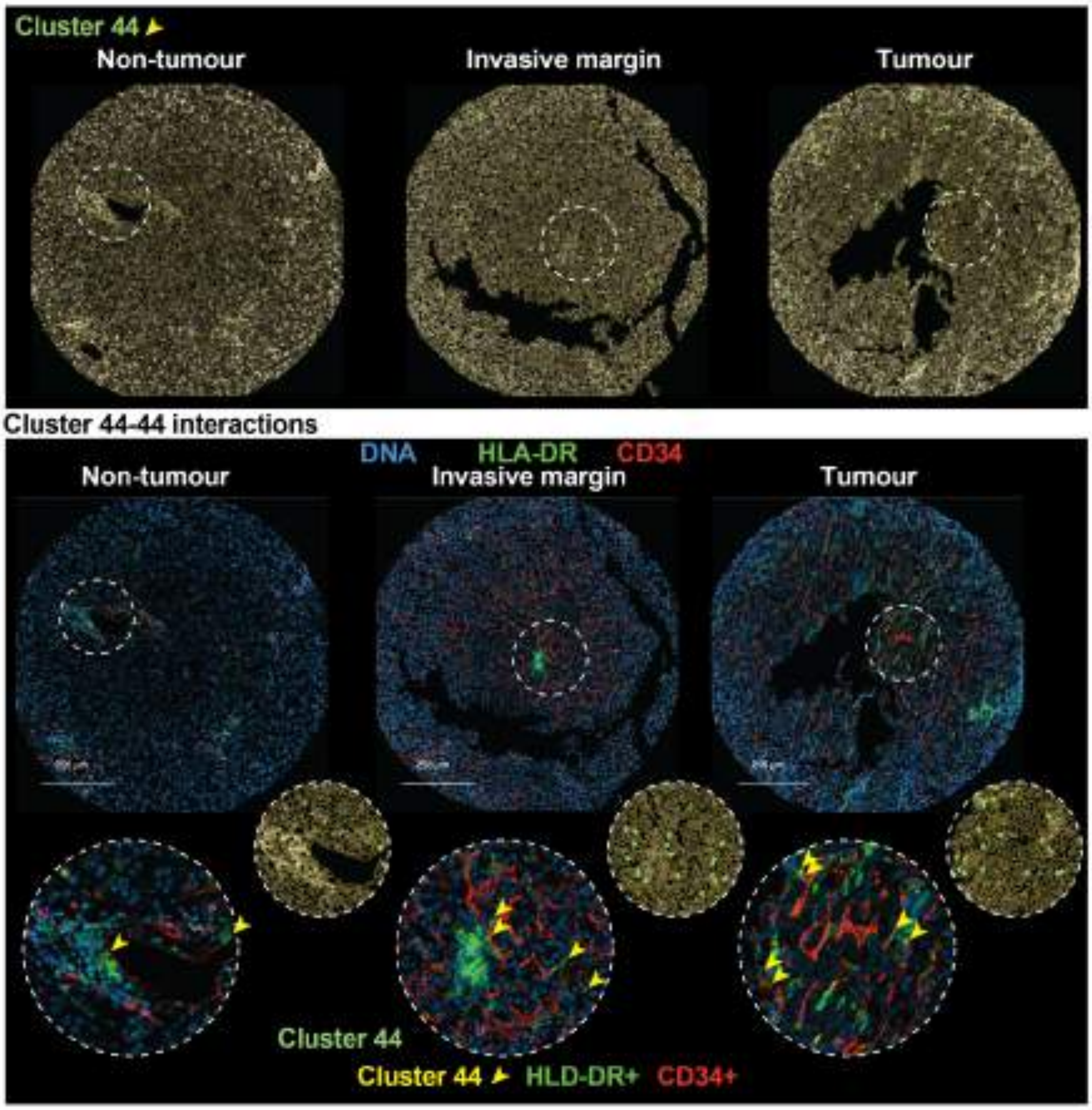
Cluster 44
Non-tumour
Invasive margin
Tumour
Cluster 44-44 interactions
DNA
HLA-DR
CD34
Non-tumour
Invasive margin
Tumour
Cluster 44
Cluster 44
HLD-DR+
CD34+

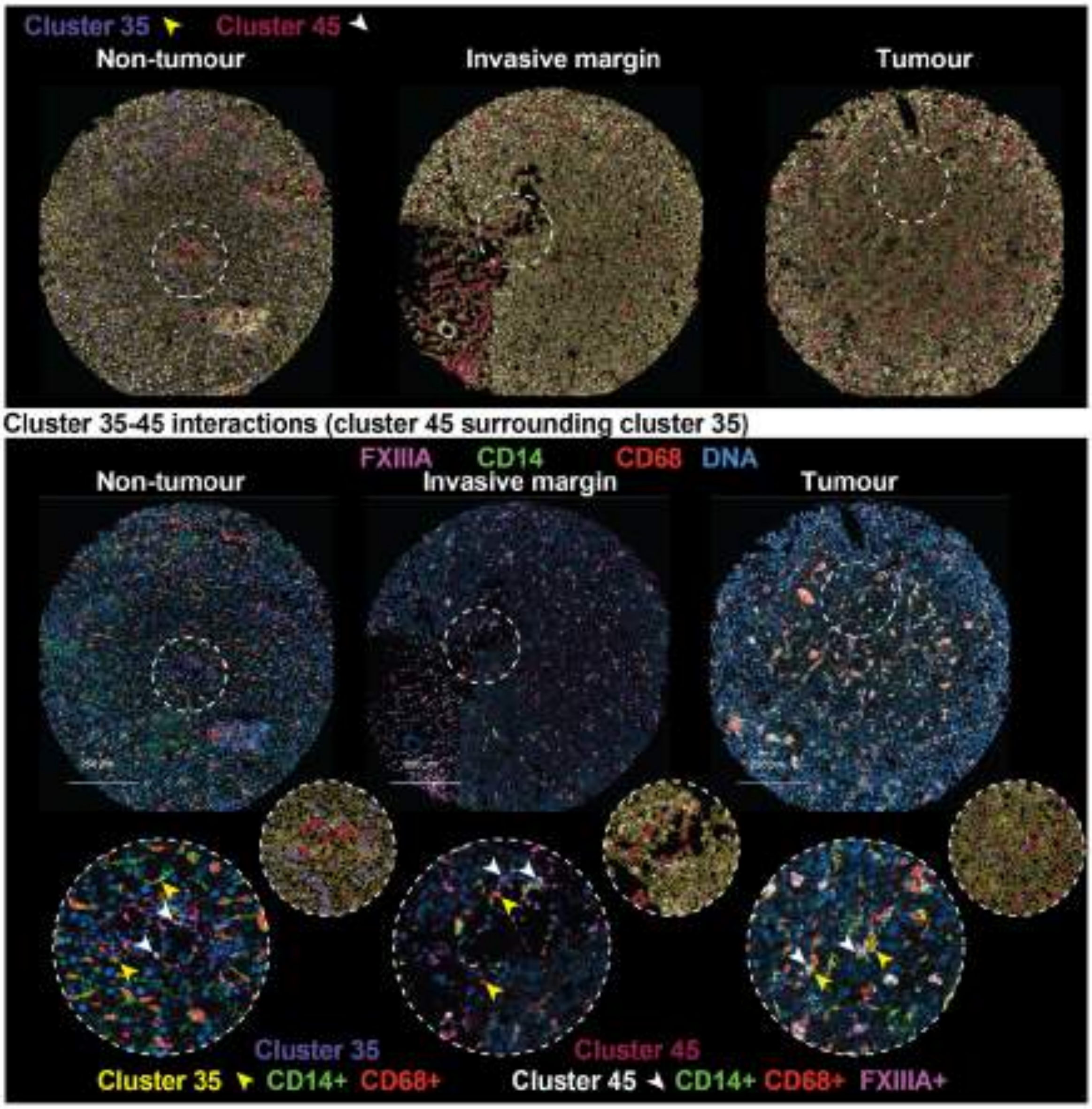
Cluster 35 Cluster 45
Non-tumour
Invasive margin
Tumour
Cluster 35-45 interactions (cluster 45 surrounding cluster 35)
FXIIIA CD14 CD68 DNA
Non-tumour
Invasive margin
Tumour
Cluster 35
Cluster 45
Cluster 35 CD14+ CD68+
Cluster 45 CD14+ CD68+ FXIIIA+

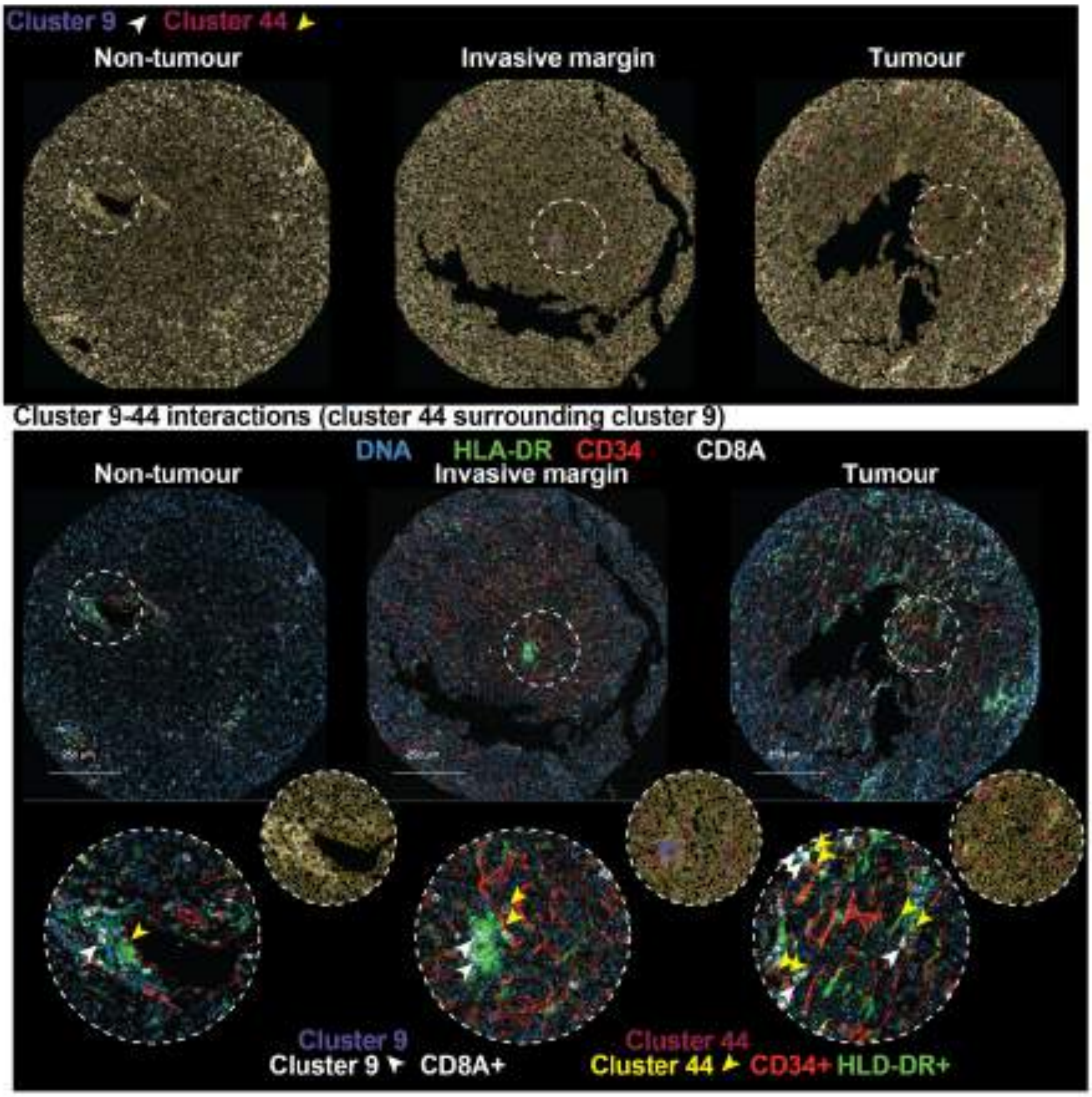
Cluster 9
Cluster 44
Non-tumour
Invasive margin
Tumour
Cluster 9-44 interactions (cluster 44 surrounding cluster 9)
DNA
HLA-DR
CD34
CD8A
Non-tumour
Invasive margin
Tumour
Cluster 9
Cluster 9 CD8A+
Cluster 44
Cluster 44 CD34+ HLD-DR+

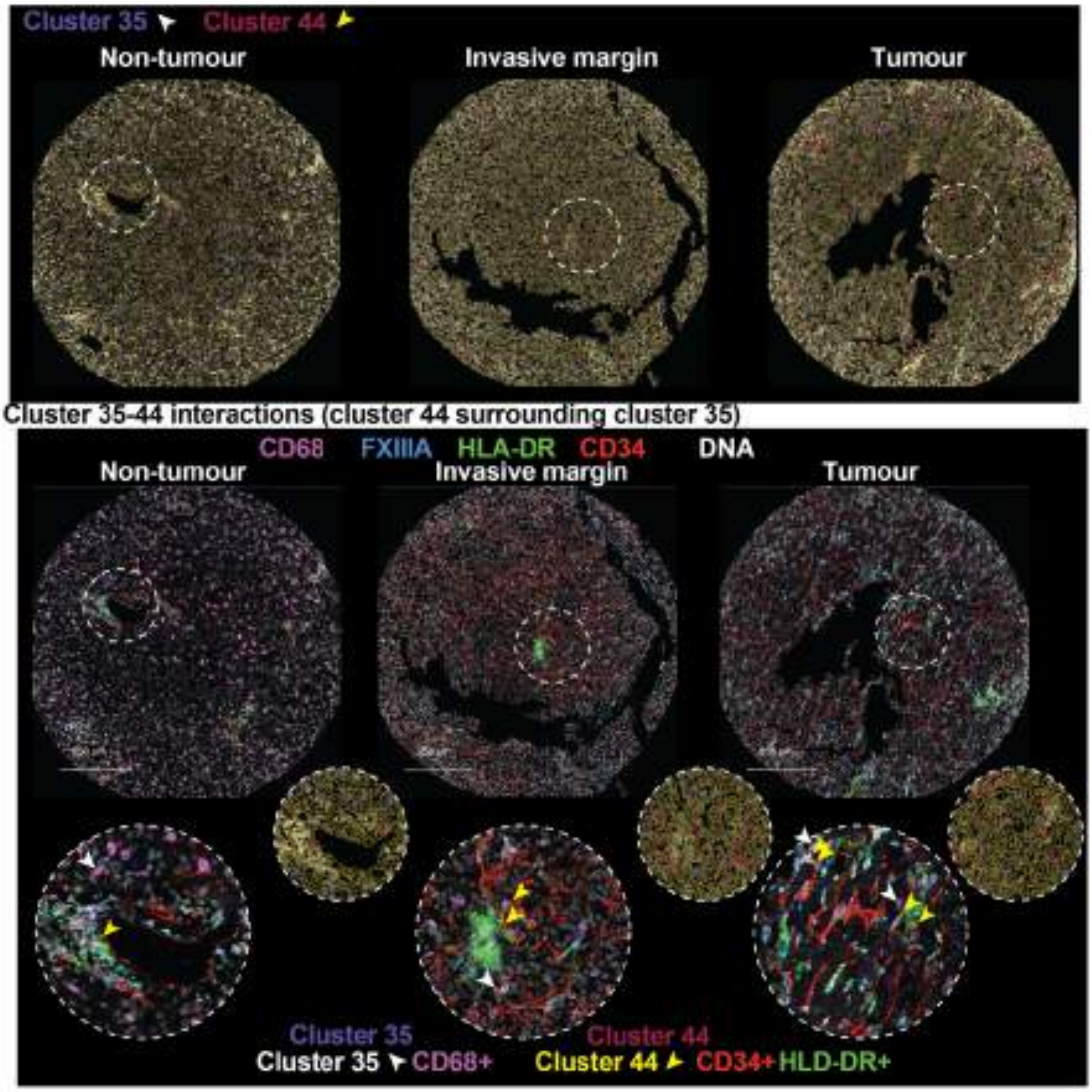
Cluster 35
Cluster 44
Non-tumour
Invasive margin
Tumour
Cluster 35-44 interactions (cluster 44 surrounding cluster 35)
CD68
FXIIIA
HLA-DR
CD34
DNA
Non-tumour
Invasive margin
Tumour
Cluster 35
Cluster 44
Cluster 35 CD68+
Cluster 44 CD34+ HLD-DR+

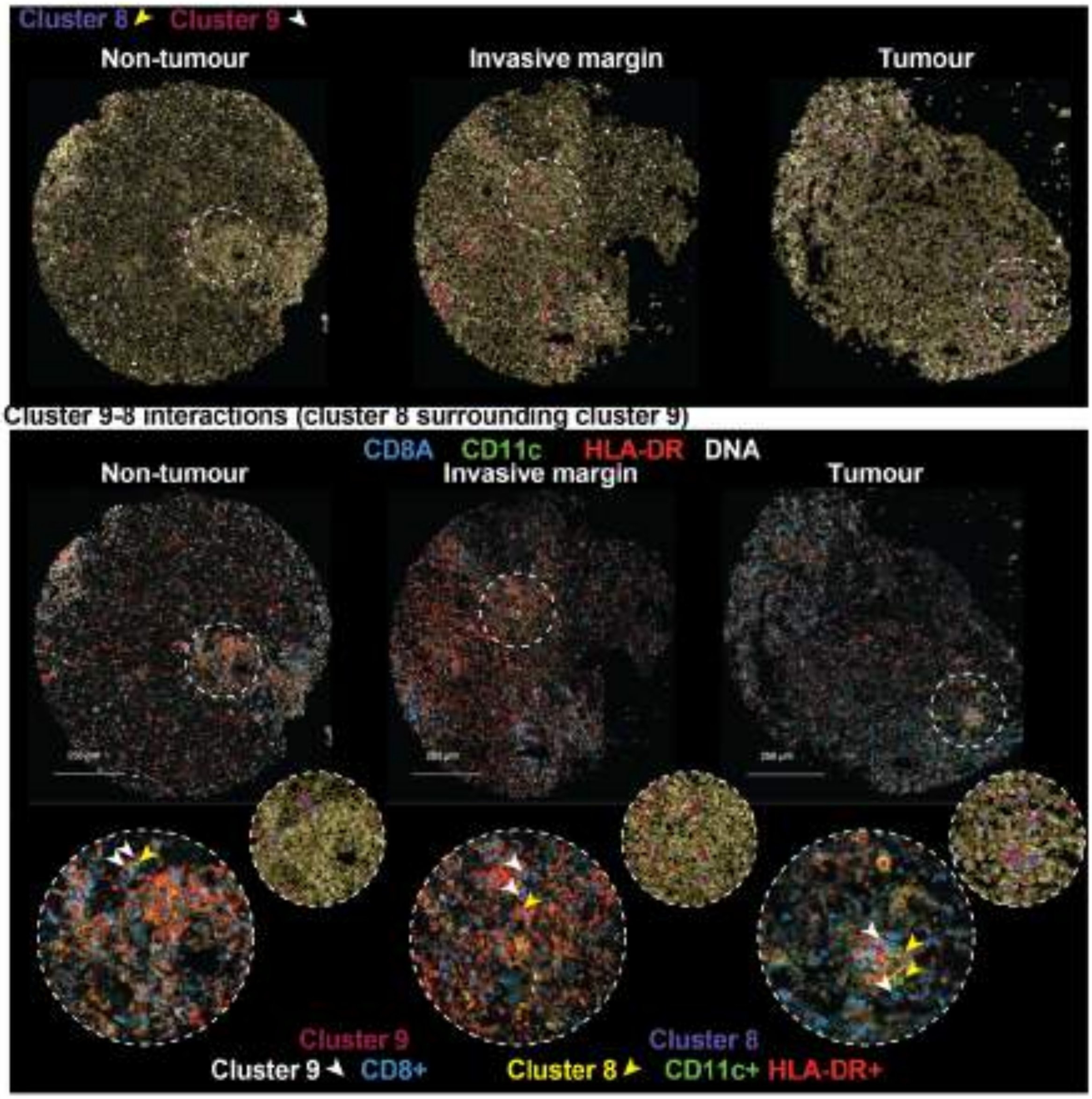
Cluster 8 Cluster 9
Non-tumour
Invasive margin
Tumour
Cluster 9-8 interactions (cluster 8 surrounding cluster 9)
CD8A CD11c HLA-DR DNA
Non-tumour
Invasive margin
Tumour
Cluster 9
Cluster 8
Cluster 9 CD8+
Cluster 8 CD11c+ HLA-DR+

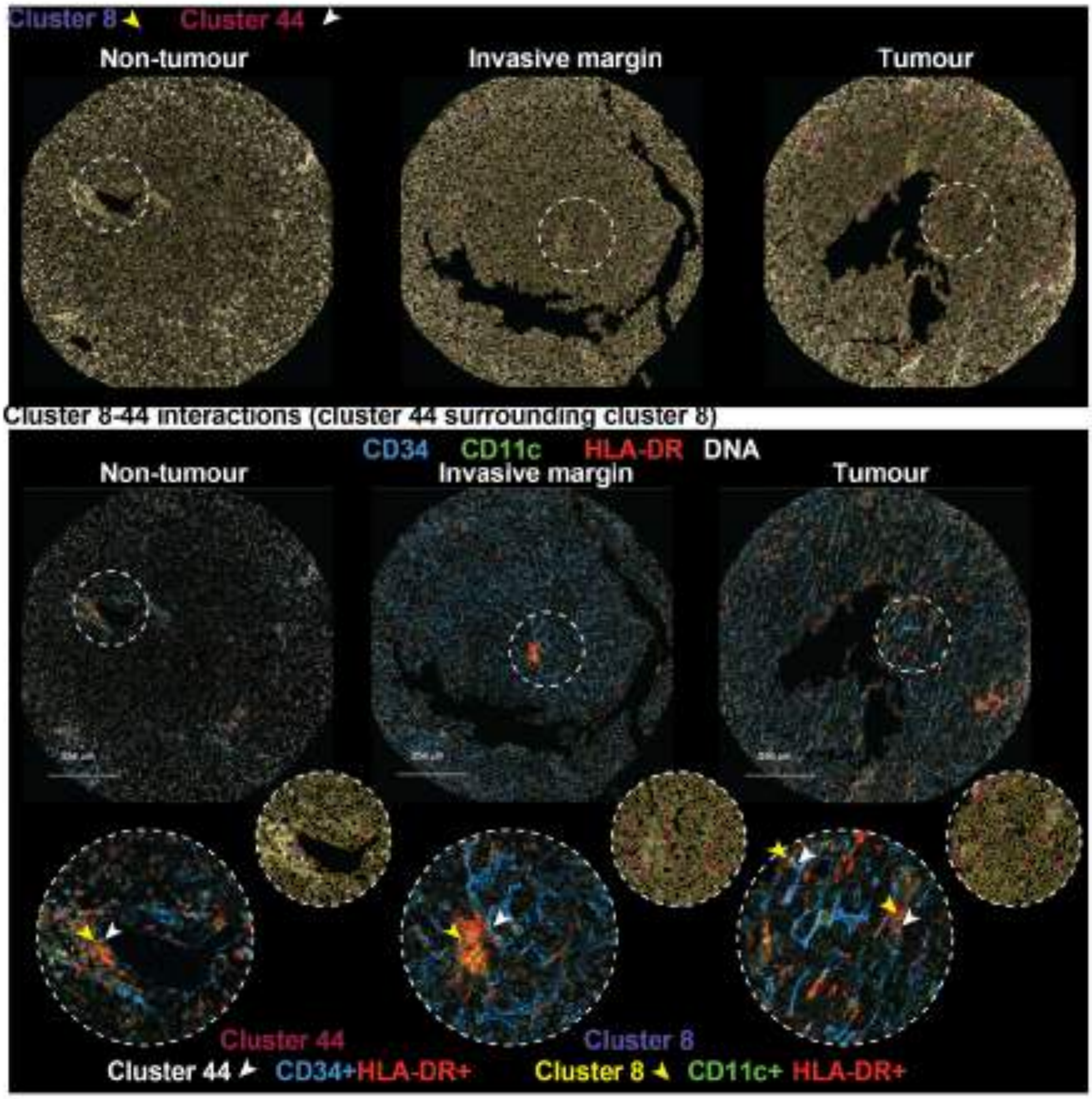

Cluster 8
Cluster 44
Non-tumour
Invasive margin
Tumour
Cluster 8-44 interactions (cluster 44 surrounding cluster 8)
CD34
CD11c
HLA-DR
DNA
Non-tumour
Invasive margin
Tumour
Cluster 44
Cluster 8
Cluster 44
CD34+HLA-DR+
Cluster 8
CD11c+ HLA-DR+

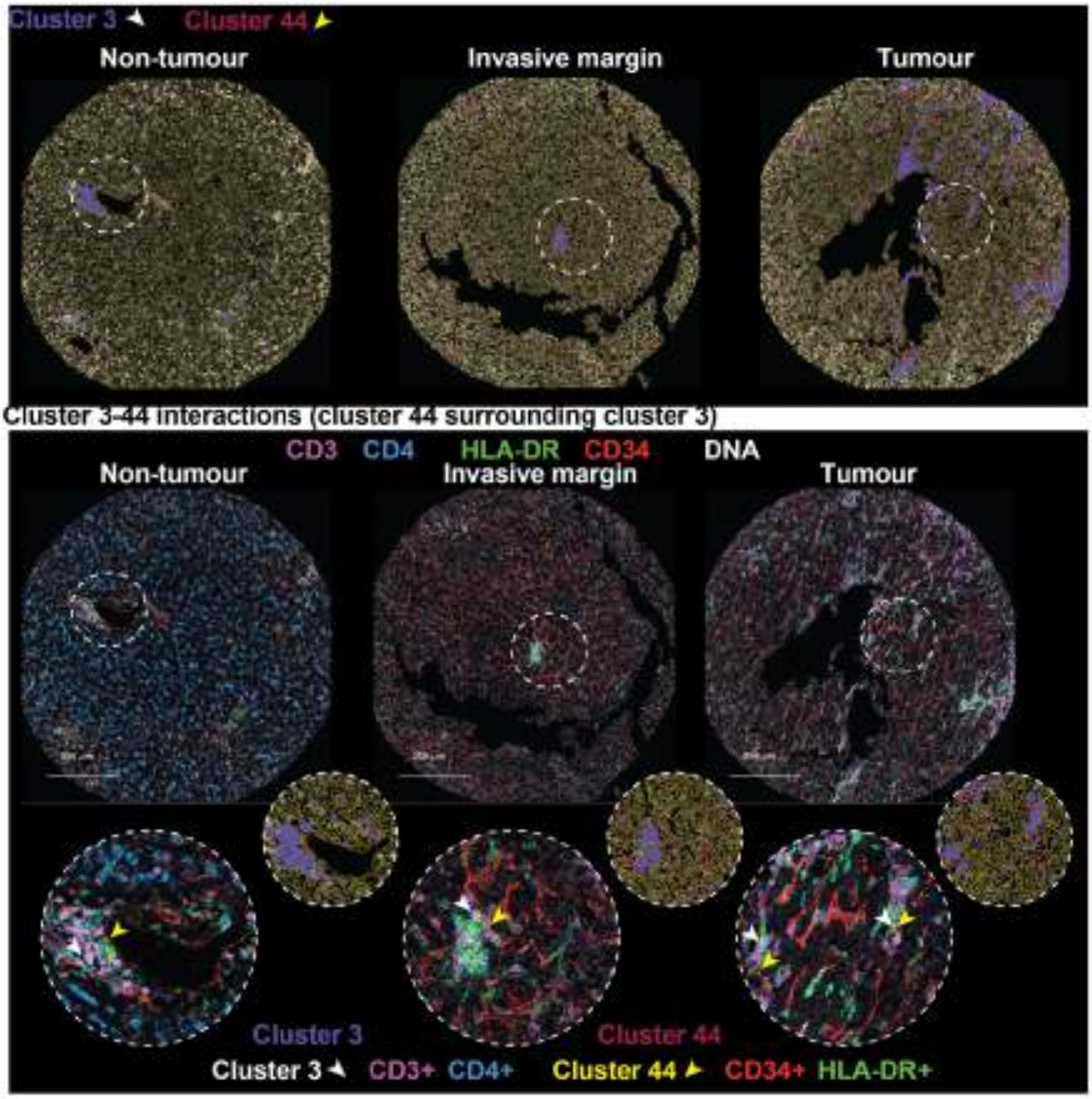

Supplementary Figure 8 – Representative images of parameters that contributed to differences between non-tumour and tumour regions. Representative images of cluster results on segmented data (green, dark blue, dark red dots). Images of imaging mass cytometry with markers and clusters as indicated.

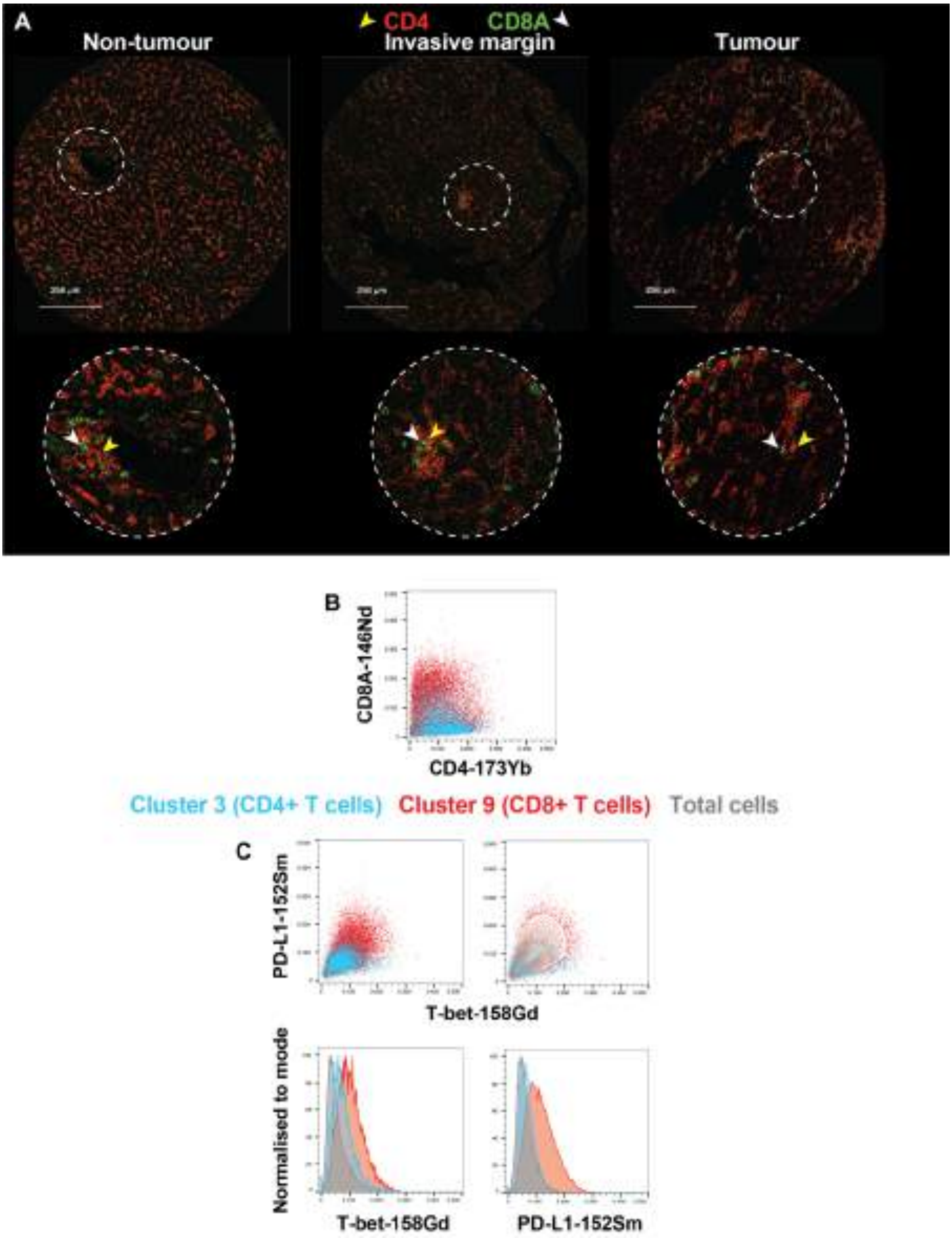

Supplementary Figure 9 –T cell marker expression. A) Representative image of CD4 (red) and CD8A (green) staining. B) Plot showing cluster 3 (blue, $CD4^+$ T cells), cluster 9 (red, $CD8^+$ T cells), and total cells (grey) against CD4 and CD8 staining. C) Plots showing cluster 3 (blue,

$CD4^+$ T cells), cluster 9 (red, $CD8^+$ T cells), and total cells (grey) for PD-L1 and T-bet expression.

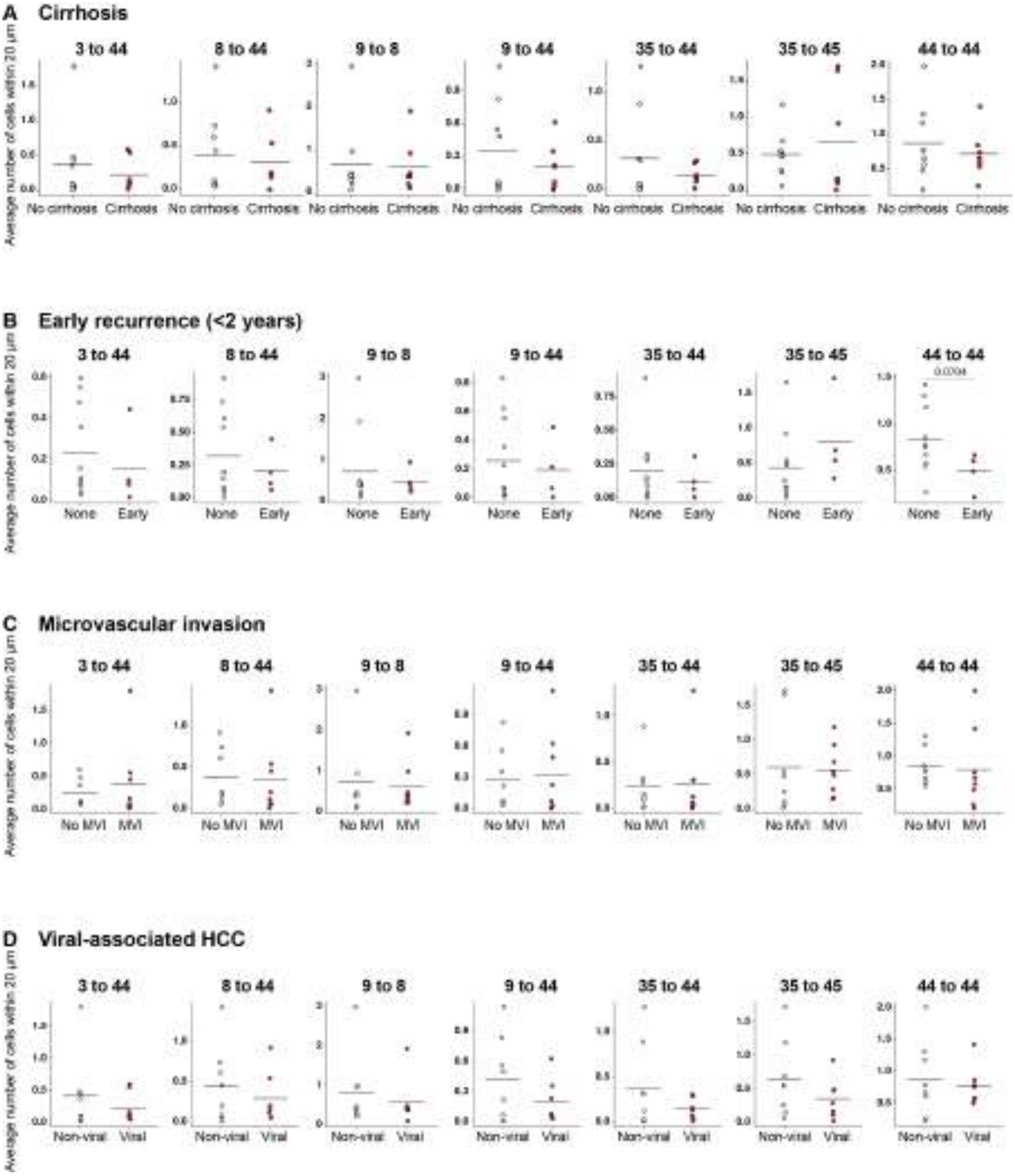

Supplementary Figure 10 – No association between clinical parameters and tumour neighbourhood interaction. Tumour neighbourhood interactions were correlated with A) cirrhosis, B) early recurrence (<2 years), C) microvascular invasion (MVI), and D) viral-associated HCC. Permutation student's t-test.

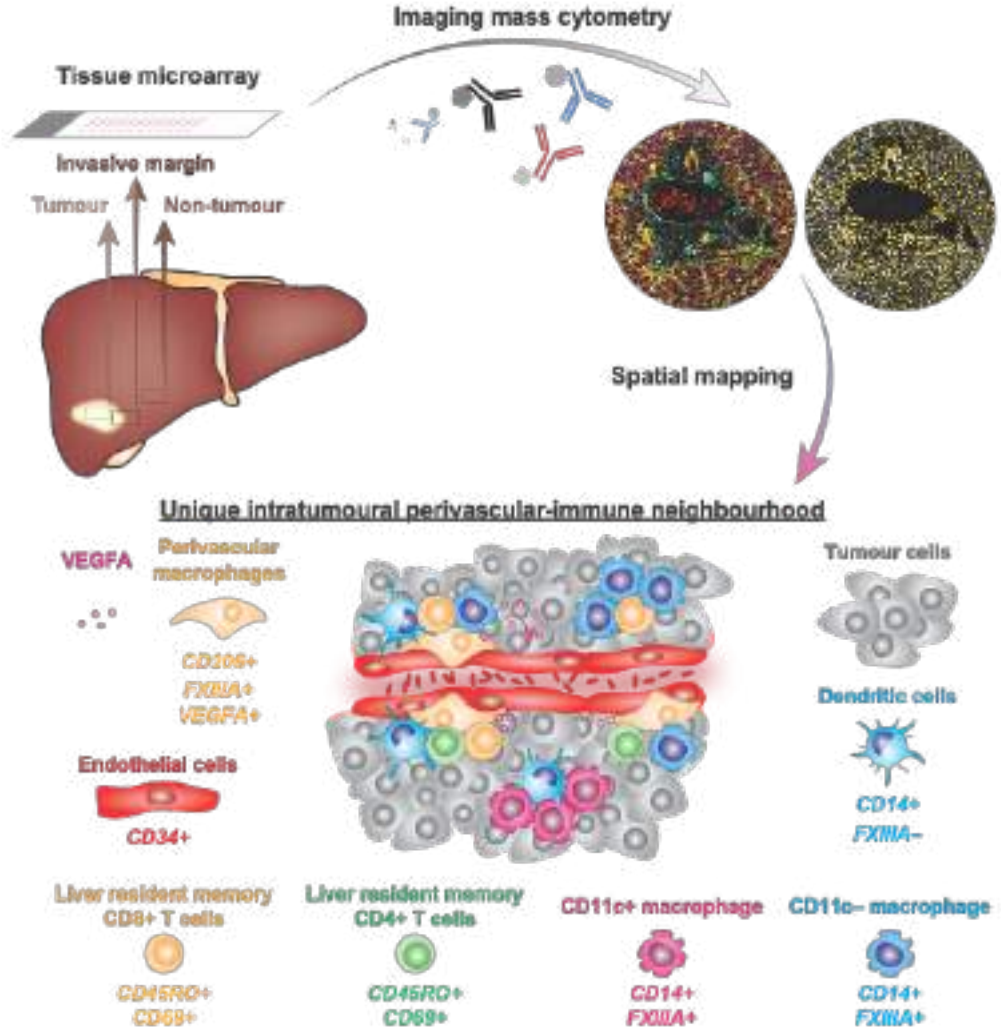

Supplementary Figure 11 – Summary figure. Liver resections were collected from patients with HCC to create a tissue microarray. Regions include non-tumour, invasive margin, and tumour. Imaging mass cytometry was done on sections with a representative image shown. Within the tumour, a unique intratumoural perivascular-immune neighbourhood was identified, featuring cells as described.

Supplemenary Table 1: Patient cohort

ID	Experiment	Aetiology	Cirrhotic	BCLC	TNM	Largest tumour size (mm)	Tumour number	MVI	Differentiation
1	IMC (TMA1)	HBV	Y	A	pT2	17	2	Y	Moderate
2	IMC (TMA1)	MASH	N	A	pT2	165	1	Y	Moderate
3	IMC (TMA1)	HBV	Y	A	pT1	40	2	N	Well
4	IMC (TMA1)	HCV, MASH	Y	A	pT1b	32	1	N	Poor
5	IMC (TMA1)	ALD	Y	A	T1bN0MX	35	1	N	Moderate
6	IMC (TMA1)	MASH	N	A	pT1b	37	1	N	Moderate
7	IMC (TMA1)	MASH	N	A	pT3	270	1	Y	Moderate
8	IMC (TMA1)	HBV	N	A	pT2	117	1	Y	Well to moderate
9	IMC (TMA2)	HCV	Y	A	pT2	30	1	Y	Moderate
10	IMC (TMA2)	HCV	N	A	pT2 Nx	170	1	Y	Moderate
11	IMC (TMA2)	ALD	Y	A	pT2	25	1	Y	Well to moderate
12	IMC (TMA2)	Unknown	N	B	T3 N0 M0	110	>3	Y	Moderate
13	IMC (TMA2)	HCV	Y	A	pT2	21	1	N	Well to moderate
14	IMC (TMA2)	HBV	N	A	pT1a	16	1	N	Moderate
15	IMC (TMA2)	MASH, ALD	N	A	pT1a	9	1	N	Well
16	IMC (TMA2)	Unknown	N	A	pT1b N0	140	1	N	Poor
17	OPAL	HBV	N	A	pT1b N0M0	150	1	Y	Moderate
18	OPAL	Unknown	Y	A	pT1b N0M0	120	1	N	Well
19	OPAL	ALD	N	A	pT2 N0M0	130	1	Y	Moderate
20	OPAL	HCV, ALD	Y	B	pT2 N0M0	45 + 18	2	N	Moderate
21	OPAL	Unknown	N	A	pT2 N0M0	160	1	Y	Poor
22	OPAL	HCV	N	A	pT4 N1 M0	45	1	Y	Poor
23	OPAL	Unknown	N	A	pT2 N0M0	180	1	Y	Moderate
24	OPAL	HBV	N	A	pT1b N0M0	71	1	N	Moderate

Supplementary Table 2: Imaging mass cytometry antibody panel

Metal	Marker	Clone	Species isotype	Company	Concentration
89Y	a-SMA	1A4	Mouse IgG2a	R&D Systems	1 μg mL^{-1}
113In	Vimentin	D21H3	Rabbit IgG	CST	1 μg mL^{-1}
139La	CD57	HNK-1	Mouse IgM	BD Biosciences	2 μg mL^{-1}
141Pr	CD20	H1	Mouse IgG2a	BD Biosciences	4 μg mL^{-1}
142Nd	CD11c	EP1347Y	Rabbit IgG	Abcam	2 μg mL^{-1}
143Nd	Foxp3-biotin	236A/E7	Mouse IgG1	eBioscience	10 μg mL^{-1}
	Anti-biotin	1D4-C5	Mouse IgG2a	DVS	1/100 dilution
144Nd	MPO	E1E7l	Rabbit IgG	CST	8 μg mL^{-1}
145Nd	E-cadherin	36/E-cadherin	Mouse IgG2a	BD Biosciences	4 μg mL^{-1}
146Nd	CD8a	D8A8Y	Rabbit IgG	CST	1.98 μg mL^{-1}
147Sm	Podoplanin	polyclonal (cat #AF3670, batch #XXO0219081)	Sheep IgG	R&D Systems	2 μg mL^{-1}
148Nd	CD16	EPR16784	Rabbit IgG	Abcam	8 μg mL^{-1}
149Sm	CD45RO	UCHL1	Mouse IgG2a	BioLegend	4 μg mL^{-1}
150Nd	CD69	EPR21814	Rabbit IgG	Abcam	0.986 μg mL^{-1}
	Anti-FITC	FIT-22	Mouse IgG1	BioLegend	4 μg mL^{-1}
151Eu	HepPar1*	OCH1E5	Mouse IgG1	Santa Cruz	2 μg mL^{-1}
152Sm	PD-L1	E1L3N	Rabbit IgG	CST	1/2000 dilution
	Cy3-donkey anti-rabbit	polyclonal (code 711-166-152, lot 122096)	Donkey IgG	Jackson ImmunResearch Laboratories	1/100 dilution
	Anti-Cy3	A-6	Mouse IgG1	Santa Cruz	4 μg mL^{-1}
153Eu	CD68	KP1	Mouse IgG1	BioLegend	2 μg mL^{-1}
154Sm	CD45	D9M8I	Rabbit IgG	Cell Signalling	3 μg mL^{-1}
155Gd	CD34*	QBend10	Mouse IgG1	Novus Biologicals	2 μg mL^{-1}
156Gd	CD86	polyclonal (cat #AF141NA)	Goat IgG	R&D Systems	2 μg mL^{-1}
158Gd	T-bet	D6N8B	Rabbit IgG	CST	6 μg mL^{-1}
159Tb	PD-L2*	D7U8C	Rabbit IgG	CST	50 μg mL^{-1}
160Gd	CD14	EPR3653	Rabbit IgG	Abcam	1 μg mL^{-1}
161Dy	FXIIIa	polyclonal (cat #SAF13A-AP, batch #AP1625-AR1)	Sheep IgG	Affinity Biologicals	2 μg mL^{-1}
162Dy	IgA*	3A5A5	Mouse IgG1	Proteintech	0.2 μg mL^{-1}
163Dy	CD11b	EPR1344	Rabbit IgG	Abcam	4 μg mL^{-1}
164Dy	SirPa	D6I3M	Rabbit IgG	Cell Signalling	4 μg mL^{-1}
165Ho	NaKATPase	EP1845Y	Rabbit IgG	Abcam	6 μg mL^{-1}
166Er	CD127	EPR2955(2)	Rabbit IgG	Abcam	4 μg mL^{-1}
167Er	CD66a	YTH71.3	Rat IgG2a	Abcam	4 μg mL^{-1}

168Er	Ki67	B56	Mouse IgG1	BD Biosciences	4 µg mL^{-1}
169Tm	CK8/18	5D3	Mouse IgG1	Leica	1/200 dilution
	AF647-donkey anti-mouse	polyclonal (code 715-606-150, lot 121469)	Donkey F(ab')$_2$ fragment	Jackson ImmunResearch Laboratories	1/100 dilution
	Anti-AF647/Cy5	CY5-15	Mouse IgG1	Sigma	4 µg mL^{-1}
170Er	CD3	polyclonal (cat #A045229-2)	Rabbit	Dako	4 µg mL^{-1}
171Yb	granzyme B	EPR8260	Rabbit IgG	Abcam	8 µg mL^{-1}
172Yb	CD206	685645	Mouse IgG2b	R&D Systems	4 µg mL^{-1}
173Yb	CD4	EPR6855	Rabbit IgG	Abcam	1 µg mL^{-1}
174Yb	HLA-DR	EPR3692	Rabbit IgG	Abcam	1 µg mL^{-1}
175Lu	CD303/BDCA2	polyclonal (cat #55427-1-AP)	Goat IgG	R&D Systems	4 µg mL^{-1}
176Yb	CD56	EPR2566	Rabbit IgG	Abcam	4 µg mL^{-1}
209Bi	DC-SIGN	polyclonal (cat # ab5715)	Rabbit IgG	Abcam	1.05 µg mL^{-1}

*Antibodies conjugated and titrated by our group. All others were conjugated by Sydney Cytometry or bought directly conjugated from Standard BioTools.

Materials and methods

Study design

Sixteen treatment naïve HCC patients who underwent liver resections were included in this study. Of these patients, twelve were male and the median age was 64.5 years (range 33-84 years). The aetiology of chronic liver disease was viral in seven patients, non-viral in eight patients, and dual viral and non-viral pathology in one patient. Nine patients were cirrhotic, and half the cases had evidence of microvascular invasion. The median largest tumour size was 36 mm (range 9-270 mm). Quantitative protein expression on single cells and spatial mapping of cellular neighbourhood interactions were performed on patient tissue samples from selected regions (non-tumour, invasive margin, and tumour).

Patients

Institutional human research ethics was obtained by the Sydney Local Health District Ethics Review Committee (HREC 2020/ETH02093). Research was conducted in accordance with both the Declarations of Helsinki and Istanbul, with written consent given in writing by all subjects. Sixteen treatment naïve patients undergoing curative resection for HCC from 2019 until 2021 at a single tertiary institution were identified from the Royal Prince Alfred Liver Biobank. Inclusion criteria was based on the availability of liver tissue (frozen or paraffin embedded) from adult (>18 years old) males and females with HCC diagnosis. Patients also required availability of clinical, imaging, and histopathology data. The formalin-fixed paraffin embedded (FFPE) tissues were retrieved from the Department of Tissue Pathology and Diagnostic Oncology and Royal Prince Alfred Hospital. Relevant clinicopathological data was obtained from electronic medical records. Clinicopathological data included age, sex, aetiology of liver disease, presence of cirrhosis, presence of microvascular invasion (MVI), and largest tumour size. Of the sixteen HCC patients, two have since begun treatment with immune checkpoint inhibitors, and one is now part of a placebo-controlled trial. Whole specimens of HCC tissues from an additional eight patients were used to assess the intratumoural spatial relationship of PVMs and endothelial cells. Further details are provided in Supplementary Table 1.

Tissue Microarray Preparation

The haematoxylin and eosin (H&E) stained sections from the sixteen patients were reviewed by a liver pathologist. One region of interest (ROI) was selected from the tumour, invasive margin, and non-tumour regions. The tumour ROI was selected based on the high abundance of tumour-infiltrating lymphocytes (TILs) on H&E stained sections, classifying these HCC regions as inflamed [79,84]. The invasive margin was defined at the border of the malignant tumour and consisted of approximately 50 % malignant and 50 % non-malignant tissue. The non-tumour region was selected away from the tumour. The exact distance between the non-tumour and tumour was variable across patient samples with no fixed diameter. In many patient samples, these areas were selected from different blocks so we are unable to attain a

fixed measurement for this distance. Three 1 mm^2 (triplicate) cores were taken from each ROI for creation of the two tissue microarrays (TMAs) (Figure 1A). Tonsils and hepatocellular adenoma were used as batch controls for the TMAs. Both TMAs were sectioned at 7 μm onto charged slides. Figure 1 provides an overview of the experimental design.

Antibody Panel

An antibody panel (Supplementary Table 2) was designed to identify various components of the HCC TME and background liver tissue, including malignant and non-malignant hepatocytes, immune cells, immune checkpoint inhibitors, cell signalling pathways, stromal cells, and structural markers. Antibodies were selected based on clones previously validated in our lab on human tissue [28]. Antibodies were then re-validated on FFPE liver and HCC tissue (Supplementary Figure 1). Antibodies that were purchased unconjugated were all tested and validated by conventional immunohistochemistry (IHC) prior to metal conjugation. Post-metal conjugation antibodies were revalidated and titrated using IMC. Pre-conjugated antibodies (Sydney Cytometry Reagent Bank) were tested and titrated for our tissue and staining conditions prior to experimental use. Antibody-metal conjugations were done using the X8 Maxpar conjugation kit as per the manufacturer's protocol (Standard BioTools).

Tissue staining for imaging mass cytometry

TMA sections were baked at 60 °C for 60 minutes prior to deparaffinisation and rehydration. Heat-induced antigen retrieval was performed by boiling the samples at 100 °C for 15 minutes in pH 9.0 antigen retrieval buffer (10 mmol L^{-1} Tris base, 1 mmol L^{-1} EDTA, 0.05 % Tween 20, pH 9.0). The TMA slides were allowed to cool down to room temperature (RT) before proceeding.

Sections were incubated with 3 % hydrogen peroxide prior to washing and blocking (Akoya Biosciences, Antibody Diluent/Block). Slides were then stained with anti-CD69 (clone EPR21814, Abcam) at RT before washing with TBS-T. Secondary OPAL-HRP (PerkinElmer) was added to the slides at RT before washing in TBS-T. Slides were stained with TSA Plus FITC (PerkinElmer) at RT before washing with TBS-T. Slides were microwave treated (as described above) before letting them cool down to RT before proceeding.

Slides were then washed in TBS-T before blocking in blocking buffer (Akoya Biosciences) for 45 minutes at 37 °C. Slides were blocked with 20 % donkey serum (in DPBS) for 10 minutes at RT. Slides were stained with anti-PD-L1 (clone E1L3N, CST) and anti-CK8/18 (clone 5D3, Leica) for 45 minutes at RT before washing in TBS-T. Slides were then stained with Cy3 anti-rabbit (711-166-152, Jackson ImmunoResearch Laboratories) for PD-L1 and Cy5 anti-mouse (715-606-150, Jackson ImmunoResearch Laboratories) for CK8/18. Slides were washed in TBS-T and DPBS. Slides were avidin/biotin blocked (Life Technologies).

Slides were then stained in anti-Foxp3 biotin (clone 236A/E7, eBioscience) before washing in TBS-T. TMA sections were incubated overnight with a metal-tagged antibody cocktail (Supplementary Table 2, including anti-FITC, anti-Cy3, anti-Cy5, and anti-biotin) at 4 °C.

TMA sections were washed in 0.1 % Triton-X (in DPBS) and DPBS. TMA sections were stained with Cell-ID Ir-Intercalator in DPBS (Standard BioTools). TMA sections were washed in deionised H_2O before being allowed to air dry at RT (Figure 1B).

Imaging Mass Cytometry

The Hyperion imaging mass cytometer (Standard BioTools) was used to acquire data from both TMA slides. A pulsed laser scanned and laser ablated the tissue at 200 Hz. IMC data files were analysed using MCD Viewer (version 1.0.560.6, Standard BioTools). MCD Viewer and histoCAT++ (version 2.2)[29] were used for visualising the images, TIFF extraction, and creating representative pseudo-colour images. (Figure 1C-D).

Multiplex Immunohistochemistry

FFPE tumour specimen blocks were sectioned at 4 µm and stained using OPAL multiplex IHC (mIHC) staining kit (Akoya Biosciences) according to optimised in-house protocols as previously described [30,31]. Tissue sections were baked at 60 °C for 60 minutes prior to deparaffinisation and rehydration. Heat-induced antigen retrieval was performed by boiling the samples at 100 °C for 15 minutes in pH 9.0 antigen retrieval buffer. Sections were incubated with 3 % hydrogen peroxide prior to washing and blocking (Antibody Diluent/Block, Akoya Biosciences). Sections were then incubated with a single purified primary antibody for 35 minutes at RT, washed, and then incubated with HRP (OPAL polymer HRP (Akoya biosciences), sheep-HRP (Invitrogen), or MACH-3 mouse 2-step (Biocare, 10 minutes/step)) for 10 minutes at RT. Sections were washed prior to incubation with OPAL fluorochromes diluted in tyramide signalling amplification (TSA) reagent (Akoya Biosciences). Antigen retrieval was repeated, and subsequent antibodies stained for as described above. At completion of all antibody staining, samples were counterstained for DAPI (Cell Signalling Technologies) prior to mounting with Prolong Diamond (Life Technologies).

Primary antibodies used were anti-HLA-DR (TAL-1B5, Abcam), anti-FXIIIA (SAF13A, Affinity Biologicals), anti-CD34 (QBEnd/10, Leica), and anti-VEGFA (VG1, Dako). Single marker staining is shown in Supplementary Figure 2.

Images (20X for quantification and 20X or 40X for representative images) from 8 HCC specimens within tumour regions were captured using the Mantra quantitative pathology imaging system in combination with Mantra Snap (version 1.0.3, Akoya Biosciences) and inForm (version 2.4.2, Akoya Biosciences) to spectrally process images. Multispectral images were exported as TIFF single images and were then imported into Fiji (version 1.53c) [32] to process representative images. HALO (version 3.6.4134, Indica Labs) was used to

perform cell segmentation, threshold markers for cell phenotyping, and spatial analysis. Proximity analysis was performed within the HALO Spatial Analysis module (Indica Labs). Phenotyped cells were registered and plotted together on a single plot. The Proximity Analysis tool was used to identify the number of $VEGFA^{+/-}$ macrophages within a proximity range of 0-100 μm of $CD34^{+}$ endothelial cells and non-endothelial cells in 20 μm increments.

Spatial data Analysis

Ilastik software (version 1.4.0b27) was used to segment single cells using the multicut method [33], as this method does not rely on nuclei (Figure 1C). Masks were created to then quantify marker expression levels for each single cell in R (version 4.2.0) [34] using 'Spectre' (version 1.0 and development version) [35] (Figure 1D).

Single cells were manually gated using FlowJo (version 10.8, BD Bioscience) to identify conventional immune cell subsets (Supplementary Figure 3). The area of each section was calculated using Fiji (version 1.53c) [32], which was then used to calculate the density of each conventional subset. Spectre was then used to quantify, for every cell, the average number of each cell subset within a 20 μm radius.

For unsupervised analysis, X-shift clustering (version 26-Apr-2018) was used [36], which identified 238 clusters (Supplementary Figure 4A). The median signal intensity of CD45 was calculated for each cluster before running z-score normalisation. Clusters with values below zero were considered $CD45^{-}$, and values above zero were considered $CD45^{+}$. The latter were divided into $CD45^{low}$ and $CD45^{hi}$ with a cut-off equal to the absolute value of the smallest value (Supplementary Figure 4B). Cells then underwent X-shift clustering within each of the three groups ($CD45^{-}$, $CD45^{low}$, and $CD45^{hi}$) resulting in 91, 36, and 18 clusters respectively. Similar clusters were then combined using the function simprof as part of the 'clustsig' R package (version 1.1) [37], generating 47 clusters (20 $CD45^{+}$ clusters and 27 $CD45^{-}$ clusters) (Supplementary Figure 4C). The spatial data were then quantified using Spectre [35], including cluster densities, average distance between each cluster and every other cluster, average number of cells within 20 μm for each cluster, and the proportions of each cluster. As each region was done in triplicate, the results were averaged so each patient tissue region had a single value for each quantification.

When analysing the $CD45^{+}$ clusters, the number of clusters with ≥ 5 cells in each region were calculated. Clusters with < 5 cells across less than a third of total regions were excluded, which removed six clusters (Supplementary Figure 4D).

Heatmaps were generated using Spectre. The median signal intensity for each cluster was calculated and then min-max scaled.

Each patient region contained thousands of parameters, so linear dimensionality reduction algorithms were used to reduce the complexity. A principal component analysis (PCA) was performed using Spectre to identify differences between the three tissue ROIs: non-tumour, invasive margin, and tumour. A PCA reduced the dimensions based on the overall variance within a given dataset, including human and experimental variability. In addition to a PCA, a

sparse partial least squares-discriminant analysis (sPLS-DA) was done [38]. An sPLS-DA reduces the dimensions based on differences between groups and is not as strongly affected by unwanted variability. The R package 'mixOmics' was used for the sPLS-DA (version 6.24.0)[39]. The sPLS-DA used leave-one-out validation, calculating the Mahalanobis distance with at least three components generated. When using all clusters, up to ten variables were selected for each component. For select immune cell neighbourhood interactions, there was no limit to the number of variables assessed for each component.

Ligand-receptor communication

Single-cell transcriptomic analyses were done using the GSE149614 dataset (https://www.ncbi.nlm.nih.gov/geo/query/acc.cgi?acc=GSE149614), which consisted of cells from paired non-tumour and tumour (8/10 patients) from newly diagnosed HCC patients [40]. These patients were pathologically confirmed and proven to have no other cancers. 7/10 patients had viral infection (five with HBV, two with HCV), 3/10 had tumour-node-metastasis (TNM) stage I, 1/10 had stage II, 2/10 stage IIIA, 2/10 stage IIIB, and 2/10 stage IV [40]. Pre-processed and pre-annotated data were used. Ligand-receptor communication between cells was calculated using the R package 'CellCall' (version 1.0.7)[44].

A second cohort of HCC patients were similarly analysed as a validation (https://data.mendeley.com/datasets/skrx2fz79n/1), which similarly consisted of cells from paired non-tumour and tumour regions from six HCC patients [41]. 6/6 had HBV infection. Cluster annotations from the first cohort were transferred to this second cohort using 'scANVI' as part of 'scvi-tools' (version 1.0.4) [42,43] (Supplementary Figure 5). Ligand-receptor communication was then similarly calculated on the transferred clusters.

Spatial transcriptomic analysis

Further confirmation was undertaken using publicly available CosMx SMI data made available by nanoString [45]. The data contained one HCC tumour (grade G3, stage II) and one normal liver tissue. Data had undergone cell segmentation and cell annotation. Data were analysed in R using vignette from nanoString [45].

Endothelial cells were defined based on pre-annotated data, consisting of central venous liver sinusoidal endothelial cells (LSEC), periportal LSEC, and portal endothelial cells. Macrophages consisted of non-inflammatory and inflammatory macrophages. *CD34*-expressing endothelial cells and *VEGFA*-expressing macrophages were identified based on transcript expression. Close cells were defined as being within 20 μm of each other. Ligand-receptor communication was quantified as described above.

Statistical tests

Statistics were calculated using packages available within R [34], using Type III Sum of Squares, unless stated otherwise. Plots were generated using R and GraphPad Prism (version 9.0.0). For the PCA, a permutational multivariate analysis of variance (PERMANOVA) was done. The data were scaled with the Euclidean distance calculated between datapoints. Permutational tests are powerful non-parametric tests that do not assume homogeneity of variance or normality of distribution [46]. 4,999 permutations (for a total of 5,000 tests) were done using all parameters to compare between groups (non-tumour, invasive margin, and tumour regions). The R package 'vegan' was used for PERMANOVA calculations (version 2.6-4)[47]. To calculate paired p-values between groups, the R package 'pairwiseAdonis' was used with Holm's correction for multiple comparisons (version 0.4.1)[48]. A similar method was used for the sPLS-DA, but only the parameters that were identified in the first two components were used in their calculation.

For the manually gated results, a Friedman test with Dunn's multiple comparison corrections was done to compared between groups (non-tumour, invasive margin, and tumour regions). For comparisons between non-tumour and tumour, a Wilcoxon test was used. These were calculated in Graphpad Prism (version 9.0.0).

For comparisons between non-tumour and tumour, a PERMANOVA with Holm's correction was used for the sPLS-DA (as above). For paired comparisons for individual parameters a permutation student's t-test was done using the perm.t.test function as part of the 'RVAideMemoire' R package (version 0.9-83)[49].

Data availability

Segmented IMC data are available on Zenodo (https://zenodo.org/records/10622397).

References

1. Sung H, Ferlay J, Siegel RL, et al. Global Cancer Statistics 2020: GLOBOCAN Estimates of Incidence and Mortality Worldwide for 36 Cancers in 185 Countries. *CA Cancer J Clin*. May 2021;71(3):209-249. doi:10.3322/caac.21660
2. Llovet JM, Kelley RK, Villanueva A, et al. Hepatocellular carcinoma. *Nat Rev Dis Primers*. Jan 21 2021;7(1):6. doi:10.1038/s41572-020-00240-3
3. Siegel RL, Miller KD, Fuchs HE, Jemal A. Cancer statistics, 2022. *CA Cancer J Clin*. Jan 2022;72(1):7-33. doi:10.3322/caac.21708
4. Amicone L, Marchetti A. Microenvironment and tumor cells: two targets for new molecular therapies of hepatocellular carcinoma. *Transl Gastroenterol Hepatol*. 2018;3:24. doi:10.21037/tgh.2018.04.05
5. Finn RS, Qin S, Ikeda M, et al. Atezolizumab plus Bevacizumab in Unresectable Hepatocellular Carcinoma. *The New England journal of medicine*. May 14 2020;382(20):1894-1905. doi:10.1056/NEJMoa1915745
6. Abou-Alfa GK, Lau G, Kudo M, et al. Tremelimumab plus Durvalumab in Unresectable Hepatocellular Carcinoma. *NEJM Evidence*. 2022;1(8)doi:10.1056/EVIDoa2100070
7. Ren Z, Xu J, Bai Y, et al. Sintilimab plus a bevacizumab biosimilar (IBI305) versus sorafenib in unresectable hepatocellular carcinoma (ORIENT-32): a randomised, open-label, phase 2–3 study. *The Lancet Oncology*. 2021;22(7):977-990. doi:10.1016/s1470-2045(21)00252-7
8. Ringelhan M, Pfister D, O'Connor T, Pikarsky E, Heikenwalder M. The immunology of hepatocellular carcinoma. *Nature immunology*. Mar 2018;19(3):222-232. doi:10.1038/s41590-018-0044-z
9. Marsh-Wakefield F, Ferguson AL, Liu K, Santhakumar C, McCaughan G, Palendira U. Approaches to spatially resolving the tumour immune microenvironment of hepatocellular carcinoma. *Ther Adv Med Oncol*. 2022;14:17588359221113270. doi:10.1177/17588359221113270
10. Aliya S, Lee H, Alhammadi M, Umapathi R, Huh YS. An Overview on Single-Cell Technology for Hepatocellular Carcinoma Diagnosis. *International journal of molecular sciences*. Jan 26 2022;23(3)doi:10.3390/ijms23031402
11. Yamaguchi S, Tomomatsu N, Kagoshima M, Okumoto T, Komatsu H. Effects of Y-24180, a receptor antagonist to platelet-activating factor, on allergic cutaneous eosinophilia in mice. *Life sciences*. 1999;64(11):PL139-44. doi:10.1016/s0024-3205(99)00024-7
12. Morse MA, Sun W, Kim R, et al. The Role of Angiogenesis in Hepatocellular Carcinoma. *Clin Cancer Res*. Feb 1 2019;25(3):912-920. doi:10.1158/1078-0432.CCR-18-1254
13. Liu K, Dennis C, Prince DS, et al. Vessels that encapsulate tumour clusters vascular pattern in hepatocellular carcinoma. *JHEP Rep*. Aug 2023;5(8):100792. doi:10.1016/j.jhepr.2023.100792
14. Yamaguchi R, Yano H, Iemura A, Ogasawara S, Haramaki M, Kojiro M. Expression of vascular endothelial growth factor in human hepatocellular carcinoma. *Hepatology*. Jul 1998;28(1):68-77. doi:10.1002/hep.510280111
15. Kaseb AO, Hanbali A, Cotant M, Hassan MM, Wollner I, Philip PA. Vascular endothelial growth factor in the management of hepatocellular carcinoma: a review of literature. *Cancer*. Nov 1 2009;115(21):4895-906. doi:10.1002/cncr.24537
16. Tian L, Goldstein A, Wang H, et al. Mutual regulation of tumour vessel normalization and immunostimulatory reprogramming. *Nature*. Apr 13 2017;544(7649):250-254. doi:10.1038/nature21724
17. Shigeta K, Datta M, Hato T, et al. Dual Programmed Death Receptor-1 and Vascular Endothelial Growth Factor Receptor-2 Blockade Promotes Vascular Normalization and Enhances Antitumor Immune Responses in Hepatocellular Carcinoma. *Hepatology*. Apr 2020;71(4):1247-1261. doi:10.1002/hep.30889
18. Shen H, Yu H, Li QY, et al. Hepatocyte-derived VEGFA accelerates the progression of non-alcoholic fatty liver disease to hepatocellular carcinoma via activating hepatic stellate cells. *Acta Pharmacol Sin*. May 4 2022;doi:10.1038/s41401-022-00907-5

19. Cheng S, Li Z, Gao R, et al. A pan-cancer single-cell transcriptional atlas of tumor infiltrating myeloid cells. *Cell*. Feb 4 2021;184(3):792-809 e23. doi:10.1016/j.cell.2021.01.010
20. Sharma A, Seow JJW, Dutertre CA, et al. Onco-fetal Reprogramming of Endothelial Cells Drives Immunosuppressive Macrophages in Hepatocellular Carcinoma. *Cell*. Oct 15 2020;183(2):377-394 e21. doi:10.1016/j.cell.2020.08.040
21. Traum D, Wang YJ, Schwarz KB, et al. Highly multiplexed 2-dimensional imaging mass cytometry analysis of HBV-infected liver. *JCI Insight*. Apr 8 2021;6(7):e146883. doi:10.1172/jci.insight.146883
22. Ho WJ, Zhu Q, Durham J, et al. Neoadjuvant cabozantinib and nivolumab convert locally advanced hepatocellular carcinoma into resectable disease with enhanced antitumor immunity. *Nature Cancer*. 2021;doi:10.1038/s43018-021-00234-4
23. Mi H, Ho WJ, Yarchoan M, Popel AS. Multi-Scale Spatial Analysis of the Tumor Microenvironment Reveals Features of Cabozantinib and Nivolumab Efficacy in Hepatocellular Carcinoma. *Frontiers in immunology*. 2022;13:892250. doi:10.3389/fimmu.2022.892250
24. Sheng J, Zhang J, Wang L, et al. Topological analysis of hepatocellular carcinoma tumour microenvironment based on imaging mass cytometry reveals cellular neighbourhood regulated reversely by macrophages with different ontogeny. *Gut*. Jun 2022;71(6):1176-1191. doi:10.1136/gutjnl-2021-324339
25. Ramachandran P, Dobie R, Wilson-Kanamori JR, et al. Resolving the fibrotic niche of human liver cirrhosis at single-cell level. *Nature*. Nov 2019;575(7783):512-518. doi:10.1038/s41586-019-1631-3
26. Ruf B, Bruhns M, Babaei S, et al. Tumor-associated macrophages trigger MAIT cell dysfunction at the HCC invasive margin. *Cell*. Aug 17 2023;186(17):3686-3705 e32. doi:10.1016/j.cell.2023.07.026
27. Li M, Wang L, Cong L, et al. Spatial proteomics of immune microenvironment in nonalcoholic steatohepatitis-associated hepatocellular carcinoma. *Hepatology*. Mar 1 2024;79(3):560-574. doi:10.1097/HEP.0000000000000591
28. Ferguson AL, Sharman AR, Allen RO, et al. High-Dimensional and Spatial Analysis Reveals Immune Landscape-Dependent Progression in Cutaneous Squamous Cell Carcinoma. *Clin Cancer Res*. Nov 1 2022;28(21):4677-4688. doi:10.1158/1078-0432.CCR-22-1332
29. Catena R, Montuenga LM, Bodenmiller B. Ruthenium counterstaining for imaging mass cytometry. *The Journal of pathology*. Apr 2018;244(4):479-484. doi:10.1002/path.5049
30. Hewavisenti R, Ferguson A, Wang K, et al. CD103+ tumor-resident CD8+ T cell numbers underlie improved patient survival in oropharyngeal squamous cell carcinoma. *J Immunother Cancer*. Jun 2020;8(1)doi:10.1136/jitc-2019-000452
31. Hewavisenti RV, Ferguson AL, Gasparini G, et al. Tissue-resident regulatory T cells accumulate at human barrier lymphoid organs. *Immunology and cell biology*. Sep 2021;99(8):894-906. doi:10.1111/imcb.12481
32. Schindelin J, Arganda-Carreras I, Frise E, et al. Fiji: an open-source platform for biological-image analysis. *Nat Methods*. Jun 28 2012;9(7):676-82. doi:10.1038/nmeth.2019
33. Berg S, Kutra D, Kroeger T, et al. ilastik: interactive machine learning for (bio)image analysis. *Nat Methods*. Dec 2019;16(12):1226-1232. doi:10.1038/s41592-019-0582-9
34. *R: A language and environment for statistical computing*. R Foundation for Statistical Computing; 2021. https://www.R-project.org/
35. Ashhurst TM, Marsh-Wakefield F, Putri GH, et al. Integration, exploration, and analysis of high-dimensional single-cell cytometry data using Spectre. *Cytometry Part A : the journal of the International Society for Analytical Cytology*. Mar 2022;101(3):237-253. doi:10.1002/cyto.a.24350
36. Samusik N, Good Z, Spitzer MH, Davis KL, Nolan GP. Automated mapping of phenotype space with single-cell data. *Nat Methods*. Jun 2016;13(6):493-6. doi:10.1038/nmeth.3863

37. Clarke KR, Somerfield PJ, Gorley RN. Testing of null hypotheses in exploratory community analyses: similarity profiles and biota-environment linkage. *Journal of Experimental Marine Biology and Ecology*. 2008;366(1-2):56-69. doi:10.1016/j.jembe.2008.07.009
38. Marsh-Wakefield F, Juillard P, Ashhurst TM, et al. Peripheral B-cell dysregulation is associated with relapse after long-term quiescence in patients with multiple sclerosis. *Immunology and cell biology*. Jul 2022;100(6):453-467. doi:10.1111/imcb.12552
39. Rohart F, Gautier B, Singh A, Le Cao KA. mixOmics: An R package for 'omics feature selection and multiple data integration. *PLoS Comput Biol*. Nov 2017;13(11):e1005752. doi:10.1371/journal.pcbi.1005752
40. Lu Y, Yang A, Quan C, et al. A single-cell atlas of the multicellular ecosystem of primary and metastatic hepatocellular carcinoma. *Nat Commun*. Aug 6 2022;13(1):4594. doi:10.1038/s41467-022-32283-3
41. Liu Y, Xun Z, Ma K, et al. Identification of a tumour immune barrier in the HCC microenvironment that determines the efficacy of immunotherapy. *Journal of hepatology*. Jan 25 2023;doi:10.1016/j.jhep.2023.01.011
42. Xu C, Lopez R, Mehlman E, Regier J, Jordan MI, Yosef N. Probabilistic harmonization and annotation of single-cell transcriptomics data with deep generative models. *Mol Syst Biol*. Jan 2021;17(1):e9620. doi:10.15252/msb.20209620
43. Luecken MD, Buttner M, Chaichoompu K, et al. Benchmarking atlas-level data integration in single-cell genomics. *Nat Methods*. Jan 2022;19(1):41-50. doi:10.1038/s41592-021-01336-8
44. Zhang Y, Liu T, Hu X, et al. CellCall: integrating paired ligand-receptor and transcription factor activities for cell-cell communication. *Nucleic Acids Res*. Sep 7 2021;49(15):8520-8534. doi:10.1093/nar/gkab638
45. nanoString. CosMx SMI Human Liver FFPE Dataset. Accessed 02/09/2023, https://nanostring.com/products/cosmx-spatial-molecular-imager/ffpe-dataset/human-liver-rna-ffpe-dataset/
46. Anderson MJ. A new method for non-parametric multivariate analysis of variance. *Austral Ecol*. Feb 2001;26(1):32-46. doi:DOI 10.1046/j.1442-9993.2001.01070.x
47. *vegan: Community Ecology Package*. 2019. https://CRAN.R-project.org/package=vegan
48. *pairwiseAdonis: Pairwise Multilevel Comparison using Adonis*. 2017. https://github.com/pmartinezarbizu/pairwiseAdonis
49. *RVAideMemoire: Testing and Plotting Procedures for Biostatistics*. 2021. https://CRAN.R-project.org/package=RVAideMemoire
50. Gide TN, Silva IP, Quek C, et al. Close proximity of immune and tumor cells underlies response to anti-PD-1 based therapies in metastatic melanoma patients. *Oncoimmunology*. 2020;9(1):1659093. doi:10.1080/2162402x.2019.1659093
51. Henriksson P, Becker S, Lynch G, McDonagh J. Identification of intracellular factor XIII in human monocytes and macrophages. *Journal of Clinical Investigation*. 1985;76(2):528-534. doi:10.1172/jci112002
52. Muszbek L, Adany R, Szegedi G, Polgar J, Kavai M. Factor XIII of blood coagulation in human monocytes. *Thromb Res*. Feb 1 1985;37(3):401-10. doi:10.1016/0049-3848(85)90069-6
53. Muszbek L, Yee VC, Hevessy Z. Blood coagulation factor XIII: structure and function. *Thromb Res*. Jun 1 1999;94(5):271-305. doi:10.1016/s0049-3848(99)00023-7
54. Adany R, Bardos H. Factor XIII subunit A as an intracellular transglutaminase. *Cell Mol Life Sci*. Jun 2003;60(6):1049-60. doi:10.1007/s00018-003-2178-9
55. Torocsik D, Bardos H, Hatalyak Z, et al. Detection of factor XIII-A is a valuable tool for distinguishing dendritic cells and tissue macrophages in granuloma annulare and necrobiosis lipoidica. *J Eur Acad Dermatol Venereol*. Aug 2014;28(8):1087-96. doi:10.1111/jdv.12290
56. Harney AS, Arwert EN, Entenberg D, et al. Real-Time Imaging Reveals Local, Transient Vascular Permeability, and Tumor Cell Intravasation Stimulated by TIE2hi Macrophage-Derived VEGFA. *Cancer Discov*. Sep 2015;5(9):932-43. doi:10.1158/2159-8290.CD-15-0012

57. Lewis CE, Harney AS, Pollard JW. The Multifaceted Role of Perivascular Macrophages in Tumors. *Cancer cell*. Jul 11 2016;30(1):18-25. doi:10.1016/j.ccell.2016.05.017
58. Lapenna A, De Palma M, Lewis CE. Perivascular macrophages in health and disease. *Nature reviews Immunology*. Nov 2018;18(11):689-702. doi:10.1038/s41577-018-0056-9
59. Wu R, Guo W, Qiu X, et al. Comprehensive analysis of spatial architecture in primary liver cancer. *Science Advances*. 2021;7(51)doi:10.1126/sciadv.abg3750
60. Wang YF, Yuan SX, Jiang H, et al. Spatial maps of hepatocellular carcinoma transcriptomes reveal spatial expression patterns in tumor immune microenvironment. *Theranostics*. 2022;12(9):4163-4180. doi:10.7150/thno.71873
61. Zhao N, Zhang Y, Cheng R, et al. Spatial maps of hepatocellular carcinoma transcriptomes highlight an unexplored landscape of heterogeneity and a novel gene signature for survival. *Cancer Cell Int*. Feb 2 2022;22(1):57. doi:10.1186/s12935-021-02430-9
62. Zhang S, Yuan L, Danilova L, et al. Spatial transcriptomics analysis of neoadjuvant cabozantinib and nivolumab in advanced hepatocellular carcinoma identifies independent mechanisms of resistance and recurrence. *Genome Medicine*. 2023;15(1)doi:10.1186/s13073-023-01218-y
63. Zhou P-Y, Zhou C, Gan W, et al. Single-cell and spatial architecture of primary liver cancer. *Communications Biology*. 2023;6(1)doi:10.1038/s42003-023-05455-0
64. Wu L, Yan J, Bai Y, et al. An invasive zone in human liver cancer identified by Stereo-seq promotes hepatocyte–tumor cell crosstalk, local immunosuppression and tumor progression. *Cell research*. 2023;33(8):585-603. doi:10.1038/s41422-023-00831-1
65. Armingol E, Baghdassarian HM, Lewis NE. The diversification of methods for studying cell–cell interactions and communication. *Nature Reviews Genetics*. 2024;25(6):381-400. doi:10.1038/s41576-023-00685-8
66. Yang M, Yang C, Ma D, Li Z, Zhao W, Yang D. Single-cell analysis reveals cellular reprogramming in advanced colon cancer following FOLFOX-bevacizumab treatment. *Front Oncol*. 2023;13:1219642. doi:10.3389/fonc.2023.1219642
67. Wang Y, Wang P, Zhang Z, Zhou J, Fan J, Sun Y. Dissecting the tumor ecosystem of liver cancers in the single-cell era. *Hepatol Commun*. Sep 1 2023;7(9)doi:10.1097/HC9.0000000000000248
68. Haniffa M, Ginhoux F, Wang XN, et al. Differential rates of replacement of human dermal dendritic cells and macrophages during hematopoietic stem cell transplantation. *The Journal of experimental medicine*. Feb 16 2009;206(2):371-85. doi:10.1084/jem.20081633
69. McGovern N, Schlitzer A, Gunawan M, et al. Human dermal CD14(+) cells are a transient population of monocyte-derived macrophages. *Immunity*. Sep 18 2014;41(3):465-477. doi:10.1016/j.immuni.2014.08.006
70. Yang L, Zhang Y. Tumor-associated macrophages: from basic research to clinical application. *J Hematol Oncol*. Feb 28 2017;10(1):58. doi:10.1186/s13045-017-0430-2
71. Li X, Yao W, Yuan Y, et al. Targeting of tumour-infiltrating macrophages via CCL2/CCR2 signalling as a therapeutic strategy against hepatocellular carcinoma. *Gut*. Jan 2017;66(1):157-167. doi:10.1136/gutjnl-2015-310514
72. Wu C, Lin J, Weng Y, et al. Myeloid signature reveals immune contexture and predicts the prognosis of hepatocellular carcinoma. *The Journal of clinical investigation*. Sep 1 2020;130(9):4679-4693. doi:10.1172/JCI135048
73. Kurebayashi Y, Ojima H, Tsujikawa H, et al. Landscape of immune microenvironment in hepatocellular carcinoma and its additional impact on histological and molecular classification. *Hepatology*. Sep 2018;68(3):1025-1041. doi:10.1002/hep.29904
74. Donne R, Lujambio A. The liver cancer immune microenvironment: Therapeutic implications for hepatocellular carcinoma. *Hepatology*. May 1 2023;77(5):1773-1796. doi:10.1002/hep.32740

75. Dong P, Ma L, Liu L, et al. CD86(+)/CD206(+), Diametrically Polarized Tumor-Associated Macrophages, Predict Hepatocellular Carcinoma Patient Prognosis. *International journal of molecular sciences*. Mar 1 2016;17(3):320. doi:10.3390/ijms17030320
76. Sia D, Jiao Y, Martinez-Quetglas I, et al. Identification of an Immune-specific Class of Hepatocellular Carcinoma, Based on Molecular Features. *Gastroenterology*. Sep 2017;153(3):812-826. doi:10.1053/j.gastro.2017.06.007
77. Zheng C, Zheng L, Yoo JK, et al. Landscape of Infiltrating T Cells in Liver Cancer Revealed by Single-Cell Sequencing. *Cell*. Jun 15 2017;169(7):1342-1356 e16. doi:10.1016/j.cell.2017.05.035
78. Zhang Q, He Y, Luo N, et al. Landscape and Dynamics of Single Immune Cells in Hepatocellular Carcinoma. *Cell*. Oct 31 2019;179(4):829-845 e20. doi:10.1016/j.cell.2019.10.003
79. Montironi C, Castet F, Haber PK, et al. Inflamed and non-inflamed classes of HCC: a revised immunogenomic classification. *Gut*. Jan 2023;72(1):129-140. doi:10.1136/gutjnl-2021-325918
80. Bartneck M, Schrammen PL, Möckel D, et al. The CCR2+ Macrophage Subset Promotes Pathogenic Angiogenesis for Tumor Vascularization in Fibrotic Livers. *Cellular and Molecular Gastroenterology and Hepatology*. 2019;7(2):371-390. doi:10.1016/j.jcmgh.2018.10.007
81. Li Z, Pai R, Gupta S, et al. Presence of onco-fetal neighborhoods in hepatocellular carcinoma is associated with relapse and response to immunotherapy. *Nature Cancer*. 2024;5(1):167-186. doi:10.1038/s43018-023-00672-2
82. Matsubara T, Kanto T, Kuroda S, et al. TIE2-expressing monocytes as a diagnostic marker for hepatocellular carcinoma correlates with angiogenesis. *Hepatology*. Apr 2013;57(4):1416-25. doi:10.1002/hep.25965
83. De Palma M, Venneri MA, Galli R, et al. Tie2 identifies a hematopoietic lineage of proangiogenic monocytes required for tumor vessel formation and a mesenchymal population of pericyte progenitors. *Cancer cell*. Sep 2005;8(3):211-26. doi:10.1016/j.ccr.2005.08.002
84. Galon J, Bruni D. Approaches to treat immune hot, altered and cold tumours with combination immunotherapies. *Nat Rev Drug Discov*. Mar 2019;18(3):197-218. doi:10.1038/s41573-018-0007-y
85. Liu K, Zhang X, Xu W, et al. Targeting the vasculature in hepatocellular carcinoma treatment: Starving versus normalizing blood supply. *Clin Transl Gastroenterol*. Jun 15 2017;8(6):e98. doi:10.1038/ctg.2017.28
86. Santhakumar C, Gane EJ, Liu K, McCaughan GW. Current perspectives on the tumor microenvironment in hepatocellular carcinoma. *Hepatol Int*. Dec 2020;14(6):947-957. doi:10.1007/s12072-020-10104-3
87. Haber PK, Castet F, Torres-Martin M, et al. Molecular Markers of Response to Anti-PD1 Therapy in Advanced Hepatocellular Carcinoma. *Gastroenterology*. Jan 2023;164(1):72-88 e18. doi:10.1053/j.gastro.2022.09.005
